# Operando high speed near infrared imaging during laser sintering of nanoparticles for time and space resolved temperature measurements

**DOI:** 10.1038/s41598-026-37445-7

**Published:** 2026-03-03

**Authors:** Jeldrik Schulte, Martin A. Schroer, Markus Winterer

**Affiliations:** Nanoparticle Process Technology, Faculty of Engineering and Cenide, University of Duisburg-Essen, Lotharstr. 1, Duisburg, 47057 Germany

**Keywords:** Thermography, Laser sintering, NIR, High-speed imaging, Thermal analysis, Nanoparticles, Engineering, Materials science, Nanoscience and technology, Optics and photonics

## Abstract

A high-speed camera equipped with a near-infrared (NIR) microscope optic is employed for spatially resolved NIR thermal imaging during laser sintering. The device enables operando temperature measurements with a temporal resolution less than 1 ms and a spatial resolution better than 10 μm. The feasibility for in situ temperature measurements is demonstrated for resonant laser sintering of TiO_2_ nanoparticles. The temperature range is calibrated at a framerate of 1,069 frames per second (fps) using a TiO_2_ reference sample. The laser sintering process is observed at framerates of up to 15,969 fps. The resulting microstructure of the TiO_2_ sample is analyzed by scanning electron microscopy (SEM) and interpreted in view of the recorded sintering temperature profiles.

**Supplementary Information:**

The online version contains supplementary material available at 10.1038/s41598-026-37445-7.

## Introduction

Laser materials processing is widely used in various industrial fields because of its versatile applications and relatively simple instrumentation of setups and handling. This is especially due to the high precision that can be achieved in both temporal and spatial resolution along with the precise control of the energy to be deposited. The emerging use of laser sintering and powder bed fusion (PBF) methods as additive manufacturing techniques of advanced materials has increased the demand for time-resolved process monitoring and operando characterization methods to gain higher control of the resulting material properties^1–9^.

A special type of laser materials processing is resonant laser sintering of nanoscaled semiconductors, for example metal oxides. It is a versatile and innovative material processing method for production of novel materials and functional components^10,11^. When using laser radiation of photon energies larger than the band gap of the green body material, fast and resonant heating is achieved at rather low total laser power^12^. Due to resonant and local heating in the small micrometer size range, extreme heating- and cooling rates are achieved.

A challenge for laser-based manufacturing methods in general, and for resonant laser sintering of nanoscaled materials in particular, remains the measurement of the material’s temperature along the processing path. Measurement techniques with exceptionally high spatial and temporal resolution as well as the ability to measure high temperatures are required to accurately determine this important parameter.

For instance, for resonant laser sintering, the resulting (micro-)structure strongly depends on the temperature-time-profile during the sintering process. Knowledge of the temperature distribution during the process, therefore, enables precise control of the product by variation of the deposited laser power. However, high temperatures (700 K – 2,000 K), short process times (milliseconds) and localized heating (hotspot size < 15 μm) pose severe challenges for measuring the process temperature.

One method, that is compatible with these conditions and thus can be used for operando measurements with complex material systems, is high-speed near-infrared micro thermography^3,13^.

Typically, in thermography, the infrared spectrum is divided into three regions: short-wavelength (SW), middle-wavelength (MW), and long-wavelength (LW) infrared, whose boundaries are, however, not sharply defined and vary across the literature. Common wavelength ranges are 8 to 15 μm for LW, 3 to 8 μm for MW, and 1.4 to 3 μm for SW^14^. Additionally, there is the near-infrared (NIR) range spanning the range from 750 nm to 1.4 μm. The specific infrared range has to be chosen^15^ based on the temperature range to be observed, as well as the material and application.

Major advantages of NIR thermography are its ability to achieve the highest temporal and spatial resolution in comparison to longer wavelength regions. Furthermore, the error in determining the emissivity of the material, an important parameter in thermography, has a smaller impact on temperature determination^16,17^. However, this wavelength range can only be effectively used for measurements at higher temperatures and the dynamic measurement range is smaller compared to longer wavelengths. Advantageously, sensors based on silicon technology can be used for NIR thermography. These are by far the most advanced sensors due to the enormous development of consumer cameras and smartphones^18^. Additionally, the availability and quality of lenses for this wavelength range are the highest due to the proximity to the visible spectrum. For example, Teyssieux et al.^19^ used a CCD camera with an optical microscope to conduct spatial highly resolved (smaller than1 µm) temperature measurements.

Consequently, the NIR range allows for the development of efficient and versatile high-temperature thermal cameras using affordable but highly advanced equipment. NIR temperature measurements with silicon based sensors have been used for some time in metal processing, such as welding^20^, forming and cutting^21,22^, or cutting^22^, and increasingly in additive manufacturing processes^23–25^ as well as in molecular beam epitaxy^26^.

Our instrument consists of a commercially available high-speed camera based on silicon technology, combined with NIR optimized microscope optics for time- and space-resolved temperature measurements during UV (resonant) laser sintering of TiO_2_ nanoparticles, which have been compacted before into pellets using a hydraulic press. The spatial resolution of the instrument is determined and a temperature calibration process of a nanoparticulate TiO_2_ pellet is described. The dynamic temperature range for a time resolution of 935 µs (1,069 frames per second) is evaluated. The temporal resolution of the instrument is used for monitoring the laser pulses generated by a fast shutter system.

The real time observation of the temperature field during resonant laser sintering provides access to the relevant process timescales and kinetics. A better insight of the resonant laser sintering process for further process optimization is obtained by additional ex situ characterization of the resulting microstructures via scanning electron microscopy (SEM).

## Results and discussion

### Temperature calibration of the NIR-camera

**Fig. 1:**
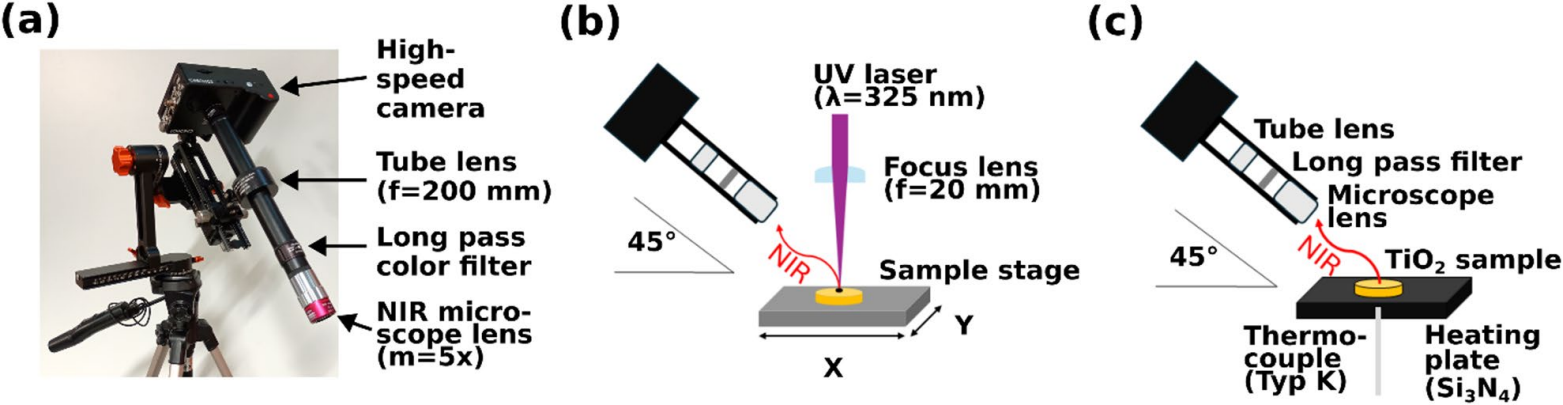
(a) Chronos 1.4 high-speed camera mounted on a micrometer adjustment slide and a gimbal tripod head. A NIR optimized microscope optic with a magnification of 5 is applied for higher spatial resolution. (b) Schematic of the laser sintering set up and the high-speed NIR imaging system. (c) Schematic of calibration process.

Figure 1 shows the fully equipped NIR camera system (a) as well as the two installations used within this work: b) resonant UV-laser (wavelength of 325 nm) sintering and c) the calibration process. Details on the equipment are given in “Methods” section. In the following, we firstly address the temperature calibration process.

In order to measure the temperature of the nanoparticulate TiO_2_ pellet during laser sintering via NIR thermography, the NIR camera system must be calibrated. This is conducted for a temperature range of 873 K to 1,173 K. The emissivity of the nano-structured, heated TiO_2_ sample is unknown, especially for the observation conditions as applied during laser sintering. Therefore, a direct temperature-sample calibration is performed under the same measuring conditions as for the laser sintering study. The sample is measured at the same distance and under the same zenith angle (45°) as during laser sintering. In general, the measurement angle has to be taken into account as the emissivity is angle-dependent, however, at zenith angles less than 45° its effect is usually rather low^27^.

The TiO_2_ pellet is heated with a ceramic heating plate and the temperature is measured using a type-K thermocouple (TC), which is placed into a blind hole in the heating plate beneath the sample position (detailed description is in the “Temperature measurement principle and calibration” section). Additionally, the apparent surface temperature is measured using a pyrometer [Fig. S. 1.] A bismuth oxide (Bi_2_O_3_) pellet is prepared and heated in a same manner as the TiO_2_ pellet to determine the temperature difference of the actual sample temperature and the measured temperature by the thermocouple [Fig. S. 2.]. Bi_2_O_3_ is used, as its melting point is in the upper middle of the calibration range and, therefore delivers a good measure of the absolute temperature value. The pellet melted completely when the TC showed a temperature of 1093 K. Although there are two different reported melting points (1,090 K^28^ and 1,098 K^29^), the experiment revealed that the deviation of the sample temperature and the TC temperature is within the uncertainty of the TC measurement. The uncertainty of the TC is given as 0.75% of the temperature.

A high-temperature calibration of a thermal camera is a sensitive procedure due to the strong non-linearity of the emitted radiation. Consequently, the calibration must be performed carefully, as systematic errors on the order of several hundred Kelvin can arise otherwise. Determining an accurate absolute sample temperature is particularly challenging, whereas relative temperature changes can be detected with high resolution owing to the exponential dependence of the emitted radiation on temperature.

With an 850 nm long-pass filter and a camera exposure time of 929 µs (1,069 fps), measurements of the TiO_2_ sample are conducted in the temperature range from 773 K to 1,173 K. The 850 nm long-pass filter is chosen to remove the contributions from visible light and UV radiation from the laser, while still covering a wide NIR spectrum at which the sensor is sensitive to maximize the recorded intensity. This enables exposure times as short as the 929 µs in the temperature range as expected during UV laser sintering. Measurements at lower temperatures are limited by the low emitted intensity in the NIR range and the dark current noise of the camera at this exposure time. While at higher temperatures (*T* > 1,249 K), the saturation range (70% of saturation limit) of the detector begins, and a nonlinear response is expected. Note, that if a different temperature range is required, the intensity and thus the accessible temperature range can be adjusted by modifying the exposure time and selecting an appropriate bandpass filter with a narrow passband frequency or by neutral density filters^23^.

**Fig. 2:**
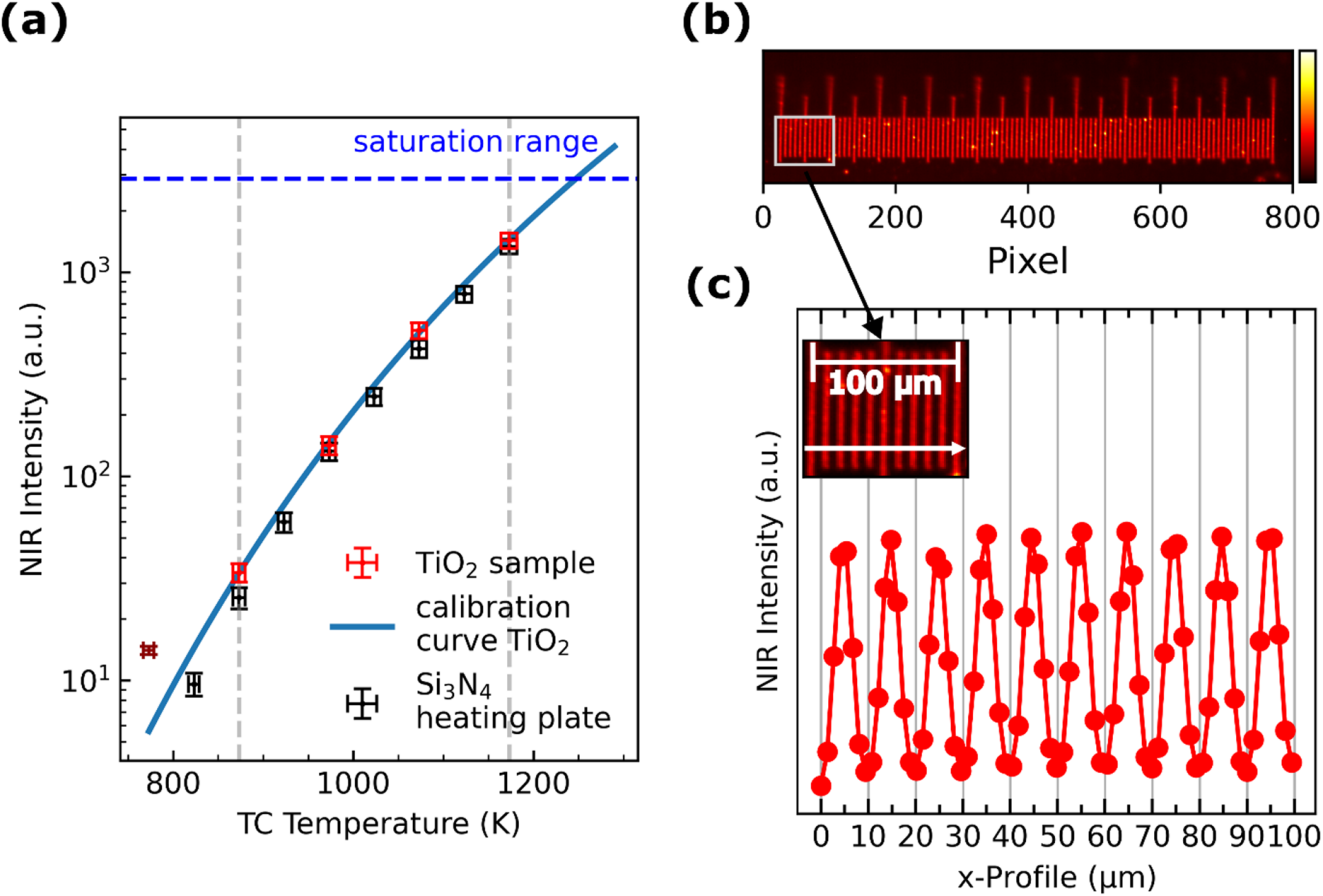
(a) Measured NIR intensities of a TiO_2_ sample at different temperatures (red markers) used for temperature calibration. A least-square algorithm is used to fit the Sakumi-Hattori function (blue, solid line). The dark red data point also belongs to TiO_2_, but is not used for calibration, as the intensity is close to the lower detection limit. Measured intensities at different temperatures of the Si_3_N_4_ ceramic heater are also shown (black markers). The temperature limits for calibration are marked as gray lines. The blue dashed line is at 70% of the full well capacity, where non-linear behavior of the detector may occure. (b) NIR (false color) image of an illuminated microscope scale. The ruler has a length of 1 mm and the distance of the bars provide a resolution of 10 μm. The colorbar indicates the measured NIR intensity (c) The intensity profile of the reflected NIR radiation. As inset is shown a magnified region of the ruler. The IFOV of 1.34 μm per pixel of the imaging system, determined by the magnification (*m* = 5x) of the microscope optics and the pixel pitch, fit accurately with the scale and the 10 μm increments are resolved.

Figure 2a presents the measured intensities at different temperatures and the resulting calibration curve for the TiO_2_ nanoparticle sample, as well as the measured intensities of the Si_3_N_4_ heating plate without sample. As for *T* = 773 K the recorded NIR intensity is close to the lower detection limit of the camera, this data point is not used for the calibration (displayed in dark red). The uncertainties of the thermocouple and of the measured intensities are displayed as horizonal and vertical error bars, respectively. The uncertainty is evaluated by the standard error of the mean intensity and the contribution of the thermocouple (details are given in the supplement). Within the temperature range of 873 K to 1,173 K, both the TiO_2_ sample and Si_3_N_4_ heating plate show an exponential increase in the measured intensity, which can be reliably fitted with the Sakuma-Hattori function. Using this calibration (explained in detail in section "Temperature measurement principle and calibration"), it is now possible to determine the temperature resolved in space and time from the recorded intensity of the NIR camera images.

### Spatial resolution

The detector of the high-speed camera has a pixel pitch of 6.6 μm, both horizontally and vertically. The NIR objective lens employed has a magnification of 5x and a focal length of 40 mm, which corresponds to a nominal spatial resolution in the focal plane of 1.34 μm/px horizontally. When the zenith angle is set to 45°, as in this study, the vertical axis is compressed by the factor of $\sqrt 2$. Hence, the vertical spatial resolution amounts to 1.90 μm/px. This defines the instantaneous field of view (IFOV) of the camera^30^. The IFOV represents the minimum field of view resolvable due to the pixel size and used optics. The intensity is normalized by the whole pixel area. Therefore, the measured temperature is underestimated if a temperature hotspot is smaller than the IFOV, as the image of the hotspot on the detector is smaller than the size of a single pixel. Since the nominal resolving power of the applied lens is 2 μm (manufacturer specification), imaging errors below 2 μm are expected.

In general, it is recommended to perform a correction using a point spread function (PSF)^31^, to achieve highest spatial resolution with a microscopic thermal camera.

The minimum field of view of a circular hotspot^32^ is defined to avoid the problem of underrating the temperature of a small hotspot. The image of a hotspot on the detector has to cover at least an area of 2 × 2 pixels. By this, at least one single pixel is fully illuminated. This leads to a minimum diameter of a hotspot of 3.8 μm for the employed camera system. The full field of view (FOV) at a framerate of 1,069 fps covers a size of 1.715 mm horizontally and 1.372 mm vertically, resolved by 1,280 × 1,024 pixels. At a zenith angle of 45° it covers a size of 1.940 mm vertically, but the FOV of the whole objective cannot be brought into focus, due to the limited depth of view. A smaller region of interest (ROI) has to be selected if higher framerates are applied due to a limited data rate of 1.4 gigapixels per second (Table 1).

An Olympus microscope calibration scale (Fig. 2b) is illuminated with a tungsten light bulb, and NIR images are captured with the same zenith angle of 45° as in the laser sintering study to evaluate the resolution capability of the camera under the applied measuring conditions. The calibration scale features 10 μm increments and the scale bar has a higher reflectivity in the NIR range compared to the background resulting in a detectible contrast. From the NIR image, an intensity profile along the scale bar direction is determined (Fig. 2c). From this, the horizontal IFOV of 1.34 μm/px is determined experimentally which is nearly identical to the calculated IFOV by pixel pitch and magnification factor of the lens. Furthermore, the intensity profile’s cross-section exhibits that the 10 μm divisions are clearly resolved. The experiment is repeated for the vertical axis [Fig. S. 3.]. For this purpose, the scale bar is rotated by 90°. The experiment reveals, that the scale can also be resolved in y-direction, but due to the zenith angle of 45°, the resolution differs by a factor of $\:\sqrt{2}$ and amounts to 1.90 μm/px.

### Operando thermal imaging during laser sintering of TiO_2_ nanoparticles

A Helium-Cadmium gas laser is used for resonant laser sintering of a pellet composed of compacted TiO_2_ nanoparticles [Fig. S. 4.]. The sample is exposed to a single laser pulse of 50 ms. Thereafter, the sample is moved to a fresh spot. This sequence is repeated for different applied laser powers, to investigate the temperature development and the impact of different laser powers on the temperature profile during resonant laser sintering of TiO_2_ nanoparticles. As second experiment, laser pulses with a constant laser power of 36 mW and varying exposure times are applied in the same manner.

**Fig. 3:**
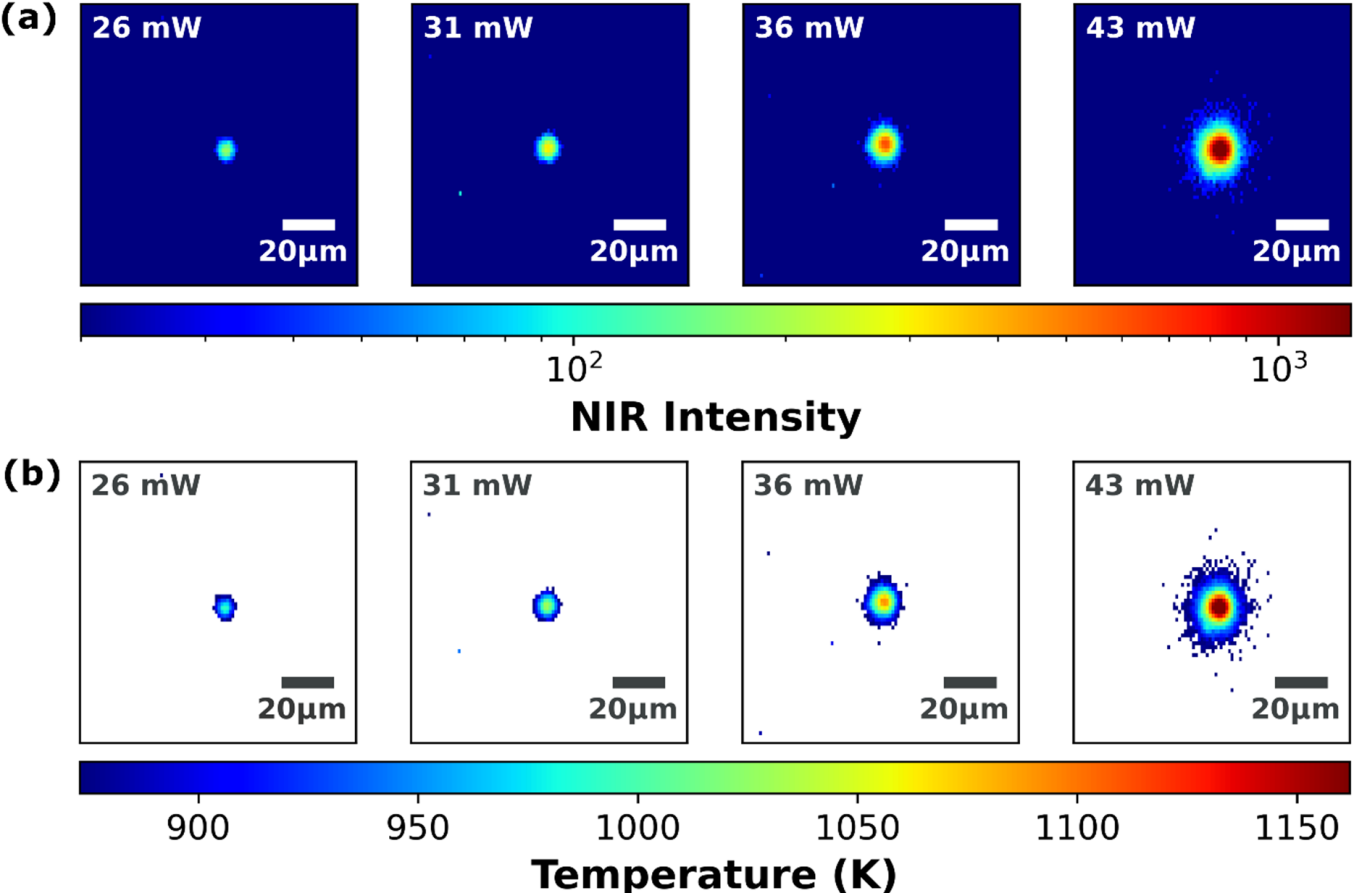
(a) False color images of the NIR intensity, measured for the laser sintering at different laser powers. The images are single frames (929 µs exposure time) taken at the middle of the laser pulse of 50 ms length. The *y*-axis of the images is stretched by a factor of $\:\sqrt{2}$ to compensate for geometric distortion due to the zenith angle of 45°. To obtain a rectangular image, fewer pixels are displayed in the *y*-direction than in *x*-direction. (b) Corresponding temperatures images (converted from the intensities using calibration). White color presents temperatures below 873 K.

Figure 3a presents NIR images during laser sintering at different laser power densities. Each false color image is a single frame with an exposure time of 929 µs, collected at the middle of the laser exposure. The selected combination of camera and lens provides spatial resolution of the sintering hotspots in the small micrometer range. The images reveal that with the selected color filter, NIR images of laser sintering can be effectively captured at a frame rate of 1,069 fps for laser powers ranging from 26 to 43 mW.

From the images, an increase of NIR intensity with rising power density is directly visible. This demonstrates that the local surface temperature is rising by enhanced power densities. In addition, the lateral expansion of the hot zone (> 900 K) increases as well. Figure 3b presents the temperature maps obtained with the calibration described in the "Temperature measurement principle and calibration" section.

It should be noted that the microstructure during the calibration process [Fig. S. 5.] develops differently than during laser sintering. This may result in a difference in emissivity, and consequently, potential deviations between the calibrated temperature and the actual sample temperature cannot be fully ruled out.

For an accurate determination of the laser sintering temperature, it has to be ensured that neither fluorescence from the sample nor reflected laser light contributes to the recorded intensity. As the used monochrome sensor of the camera is principally sensitive from the UV region up to the wavelength of 1,100 nm, the optical system needs to suppress these contributions sufficiently. The combined transmittance of the used optical components (long pass filter, tube lens and microscope optic) is about 5·10^− 10^ (manufacturer specifications) for the wavelength of 325 nm. Thus, in this case the contribution of diffuse reflected laser light is negligible due to the relatively low total laser power, the high absorption of the porous powder sample, the short camera exposure time (< 1 ms), and the perpendicular incidence (90°) of the laser beam on the surface. Nonetheless, if laser systems with higher laser power and weakly absorbing samples are used, it needs to be assessed whether reflections distort the measurement and multiple optical filters may be needed to increase the optical density.

Fluorescence measurements of TiO_2_^33,34^ reveal that the wavelengths of the fluorescence spectrum are smaller than 700 nm when excited by UV light. Consequently, the used 850 nm long pass filter strongly attenuates the fluorescence. In the Supplementary Information, an experiment [Fig. S. 6. and Fig S. 7.] is presented which demonstrates that the employed long-pass filter effectively suppresses these contributions.

**Fig. 4:**
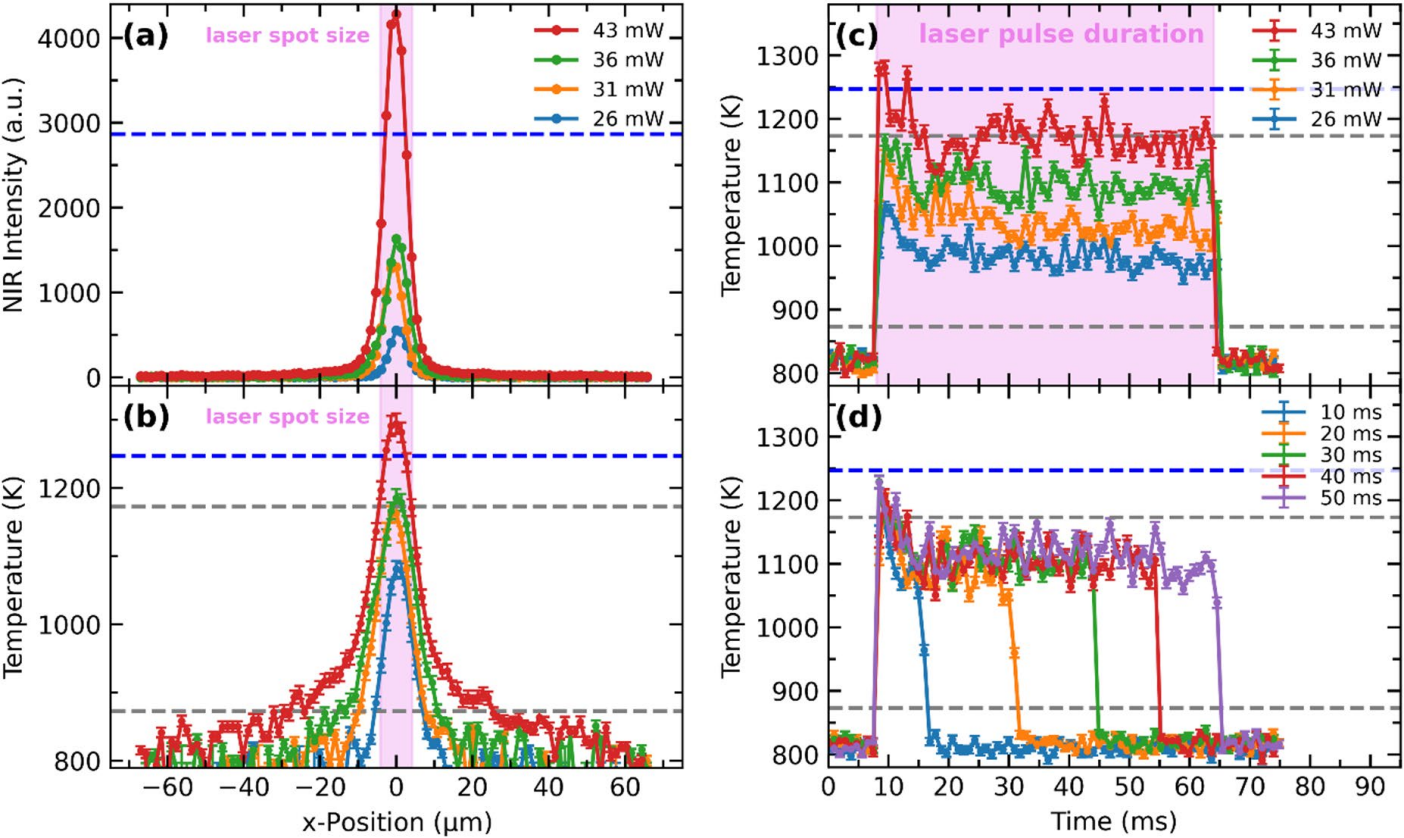
(a) NIR profiles of the sintering hotspot for different laser powers. Shown are the 80 pixels of the image row containing the image of the laser beam center. The blue dashed line is at 70% of the full well capacity, where non-linear behavior of the detector may occure. (b) The corresponding temperature profiles, obtained from calibration. The grey lines indicate the lower and the upper temperature limit of the calibration for this framerate (1,069 fps). (c) Time evolution of temperature for different power densities during laser sintering of TiO_2_ powder and constant laser pulse length of 50 ms (marked in violet). It is shown the temperature of the MFOV (3 × 3 px) at the pixel of maximum intensity (d) Time evolution of the temperature for different pulse lengths and constant laser power (*P* = 36 mW).

Line profiles of the laser sintering hotspots are shown in Fig. 4a. Here, the NIR intensities of the image row containing the pixels of maximum intensity are displayed. The profiles of the hotspots reveal a Gaussian-like temperature distribution. It is clearly visible that the NIR intensity increases with rising power density. Even small changes of the laser power of 5 mW induce an increase in the measured NIR intensity due to an increase in the surface temperature. The calculated minimum achievable spot size of the laser beam on the sample surface has a diameter (1/e²)^35,36^ of approximately 8.3 μm. The calculation is performed for a multimodal, Gaussian-like shaped laser beam, the 20 mm focus lens and the laser parameters provided and tested by the manufacturer. The calculated spot size is in good agreement with the experimentally determined spot size diameter of approximately 9 μm^10^ for the laser system and focus length. The lateral extent of the hot zone is only slightly larger than the diameter of the focused laser beam of 9 μm. The corresponding temperature profiles (Fig. 4b) reveal a steep temperature gradient at the edge of the laser spot. At the highest power, an additional, less steep temperature gradient can be observed at the edge of the laser beam. This is attributed to heat accumulation in the material, as the heat conduction is relatively low due to the porous, nanoparticulate structure of the green body. For lower power levels, this contribution is difficult to distinguish due to the limited measurement range at low temperature.

Figure 4c shows the temporal development of the temperature during laser irradiation of the TiO_2_ pellet with different laser powers. Herein, the MFOV temperature (mean of 3 × 3 pixel array) with the most intense pixel of each frame, corresponding to the hotspot of sintering, is plotted against time. For all laser powers, there is a rapid temperature increase in the first millisecond of laser exposure. The temperatures remain slightly elevated at the beginning of the laser irradiation (~ 10 ms), while thereafter sinking to relatively constant levels. The temperatures remain at these levels until shutter closing. After shutter closing, the temperature decreases rapidly within less than 2 ms to a temperature below the measurement range of 873 K. The high initial temperature can be explained by the resonant absorption and low thermal conductivity of the unsintered, porous green body. As a result, the absorbed energy remains highly localized leading to a higher absolute surface temperature. Density increases during sintering. This densification enhances local thermal conductivity, leading to a reduction in surface temperature^12^, apparently within several milliseconds.

The temperature evolution during the laser sintering of the TiO_2_ powder bed with different pulse length at fixed laser power of 36 mW is presented in Fig. 4d. Again, all profiles exhibit a steep initial temperature increase within the first millisecond of irradiation. Within the first ten milliseconds, the temperature decreases to a relative constant level (*T* = 1,100 K), revealing the same temperature behavior as before. For all pulse lengths, the temperature rise and the temperature level during irradiation remain at the same level.

In summary, the surface temperature can be controlled by laser power, whereby there is a temperature elevation in the first ten milliseconds upon laser irradiation. The holding time, during which the temperature remains at the same level, can be controlled by the laser shutter opening time. In combination, these two parameters allow for setting up a predefined temperature-time-profile of the sintering process and thus the potential control of the resulting microstructure of the material^37,38^.

The experimental setup also allows for fast probing and diagnostics of the response of the optical shutter used for laser pulse creation, which is crucial for advanced time-resolved studies. The supplier of the SH05 single blade shutter specifies a typical opening time of 4.3 ms, during which it opens from 10% to 90%, and a closing time of 6.5 ms, in which it closes from 90% to 10%. However, the laser beam size at the shutter position (1/e² diameter of 1.56 mm) is significantly smaller than the shutter aperture of 127 mm, which leads to a reduction in time of shutting the laser beam. If the motion of the shutter blade is assumed to be uniform in between these intervals, the time for a full laser release is calculated as 90 µs, and the time for full closure of the laser is calculated as 130 µs. The programmed pulse length, however, does not correspond exactly to the experimentally observed intensity trace, as can be determined from the temperature trace. For a ‘10 ms’ pulse a slightly shorter (8 ms) exposure is observed, whereas ‘20 ms’ to ‘50 ms’ pulses are 4 to 6 milliseconds longer than the nominal pulse length.

**Fig. 5:**
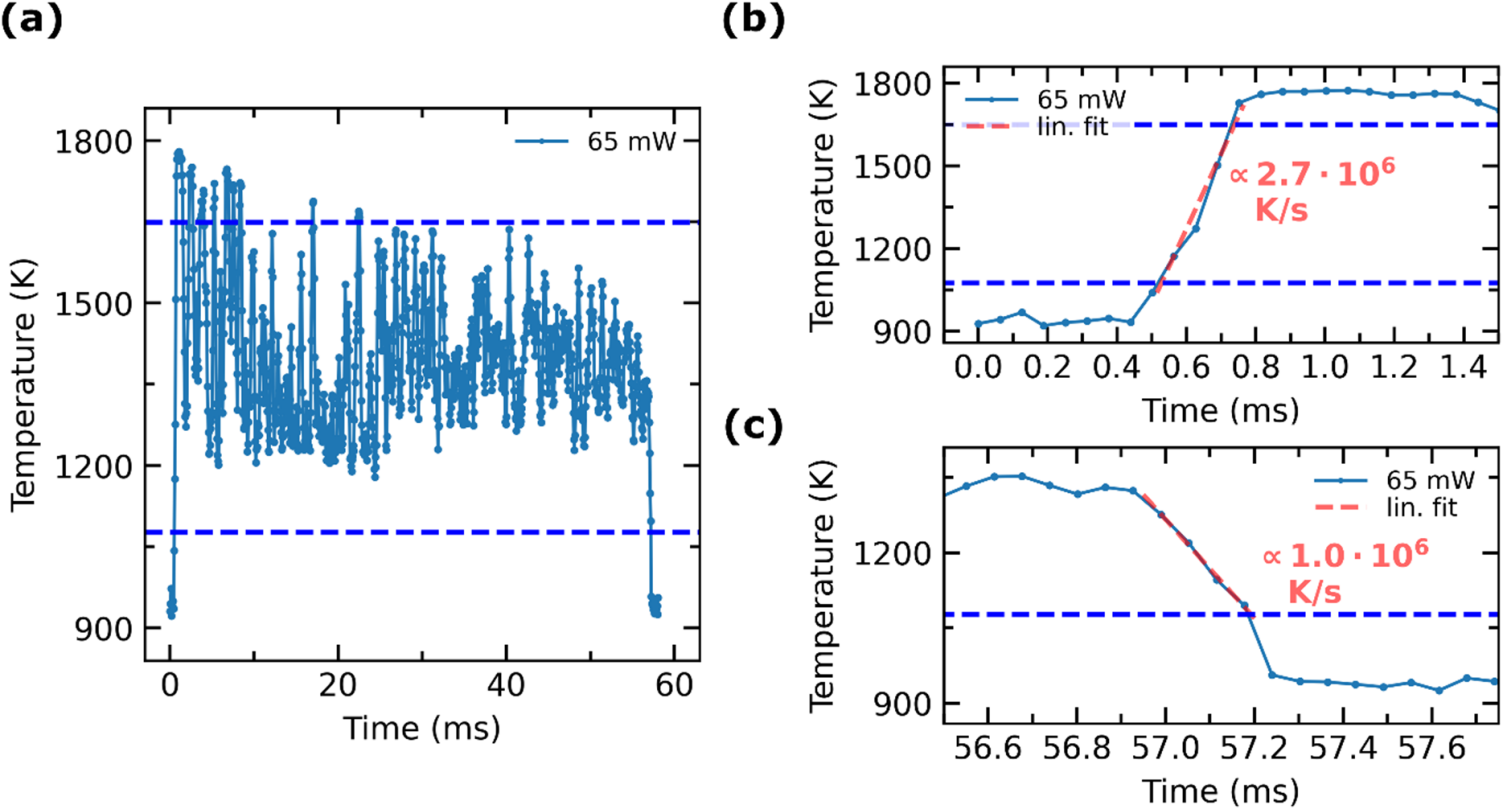
Estimated temporal development for increased laser power of 65 mW. NIR images are recorded with a frame rate of 15,969 fps. Temperature is determined from the NIR images with the MFOV (3 × 3 pixel) around the hottest pixel and by extrapolation of the calibration curve, shown in [Fig. 2a] Before extrapolation, the recorded intensities are scaled by the ratio of the exposure times. The temperatures which are outside of the blue lines are not reliable, because they are outside the assumed dynamic range of the detector. The upper blue line marks 70% of the full well capacity, above which a non-linear response of the detector may occur. The lower blue line is at the same digital number as the lowest calibration point at 1,069 fps. (a) The temporal development of the temperature during the whole laser pulse of 50 ms. (b) Temperature increase at the beginning of the laser irradiation and the temperature decrease (c) after laser irradiation. Marked are the linear approximations of the heating- and cooling rate.

Measurements with increased frame rate of the camera of 15,969 fps and an exposure time of 59.5 µs are conducted, to improve the resolution of the steep temperature gradient over time. Due to the shorter exposure time at this high frame rate, more flux is needed for a sufficient signal to noise ratio. Thus, the laser power is increased to 65 mW to reach higher temperatures and, therefore, higher exitance. Even at frame rates as high as 15,969 fps, spatially well-resolved NIR images during the sintering process are obtained, in which the development of the thermal expansion is exhibited [Fig. S. 8.]. At this frame rate, the temperature increase during the first millisecond as well as the subsequent cooling is observable with sufficient time resolution. The surface temperature which is reached at this laser power exceeds the calibration range and the calibration set up does not exhibit temperatures above 1223 K. Thus, the temperatures are estimated by extrapolation of the determined calibration curve (Fig. 2a). It has to be noted, that due to the non-linear behavior of the radiated emission an extrapolation of such a temperature span can lead to inaccuracies. Additionally, to the non-linear emission, the saturation behavior of the camera may also vary non-linearly when the exposure time is shortened. Thus, for an accurate temperature determination, a calibration has to be performed with the same exposure time and temperatures as in the actual experiments. However, the used high-speed camera showed a linear dependency between the exposure time and the recorded digital level [Fig. S. 9.] over a wide range of exposure times. Before the conversion of the NIR intensities into temperatures was carried out with the extrapolated calibration curve, the recorded intensities were scaled by the ratio of the exposure times (929 µs / 59.5 µs). The resulting temperature-time profile (Fig. 5a) reveals a similar pattern as the profiles at lower laser powers, but at the elevated temperatures at the beginning of irradiation the intensity significantly exceeds the saturation range of the detector (assuming the saturation range starts at 70% of the digital full well capacity), which means that the actual surface temperature is expected to be higher than the determined temperature. The saturation in the beginning considered, the estimated temperatures exhibit a realistic range, as a finite element simulation^12^ shows, that temperatures up to 2,000 K could be reached for laser powers of 60 mW for resonant laser sintering of a porous green body.

**Fig. 6:**
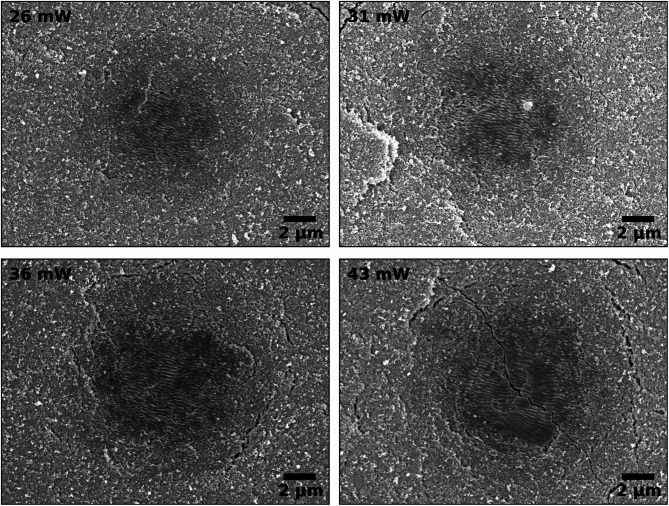
SEM images of the sintering spots at the surface of the TiO_2_ powder bed after laser irradiation with different laser powers. The sintered area in the middle of the laser beam is clearly distinct from the nanoparticulate powder bed (green body) at the edges of the images.

At a power of 65 mW, larger fluctuations occur in the temperature-time profile than for the lower laser powers (cf. Fig. 4). A possible explanation of the recorded temperature fluctuations (e.g. between 10 ms and 22 ms) during the holding time is suggested by the SEM image [Fig. S. 10.] of the structure formed after sintering. The SEM image shows a distinct indentation in the size regime of the laser spot (8 μm) and a shape that is similar to the Gaussian-shaped laser beam, whereas the SEM images of the sintering spots at lower laser power (Fig. 6) do not show indentations. This strongly implies that material has been ablated during the irradiation with a laser power of 65 mW. Ablation during the laser irradiation could explain sudden temperature drops. After the material is removed, new unsintered nanoparticles resonantly absorb the laser beam, leading to a subsequent rapid temperature increase. Additional measurements of the direct laser beam are performed using a photodiode at 25 kHz [Fig. S. 11. - S13.], to exclude the possibility that the fluctuations in the NIR signal originate from variations in the laser intensity. These measurements did not show any laser intensity variations that could account for the observed fluctuations.

The estimated heating rate is in the order of 2.7 10^6^ K/s (Fig. 5b), determined by linear approximation of the temperature increase within the measurement range, whereas the estimated temperature decrease (Fig. 5c) is in the order of 1.0 10^6^ K/s.

### Resulting microstructure

Figure 6 shows SEM images of laser sintering spots at the TiO_2_ powder bed with different power densities. These sintering spots shown in the SEM images correspond to the temperature curves presented in Fig. 4c.

The sintered surface area is clearly distinct from the nanoparticulate green body. In the laser spot, a strong densification of the surface and grain growth are observable. At powers of 26 mW and 31 mW, only a few pores are visible, mainly at the edges of the laser spot. This indicates that nearly full densification can be reached at these power levels. At 36 mW and 43 mW, intergranular pores begin to form. However, the grain structure and the grain boundaries are superimposed by laser induced periodic ripples for all laser powers, which probably occur due the polarized nature of the laser beam^39^ and have a periodicity of the order of the laser wavelength of 325 nm. It is evident that the sintered area increases with increasing power from 26 mW until 36 mW. At 43 mW, a significant increase in temperature-induced cracks in the sample becomes visible.

**Fig. 7:**
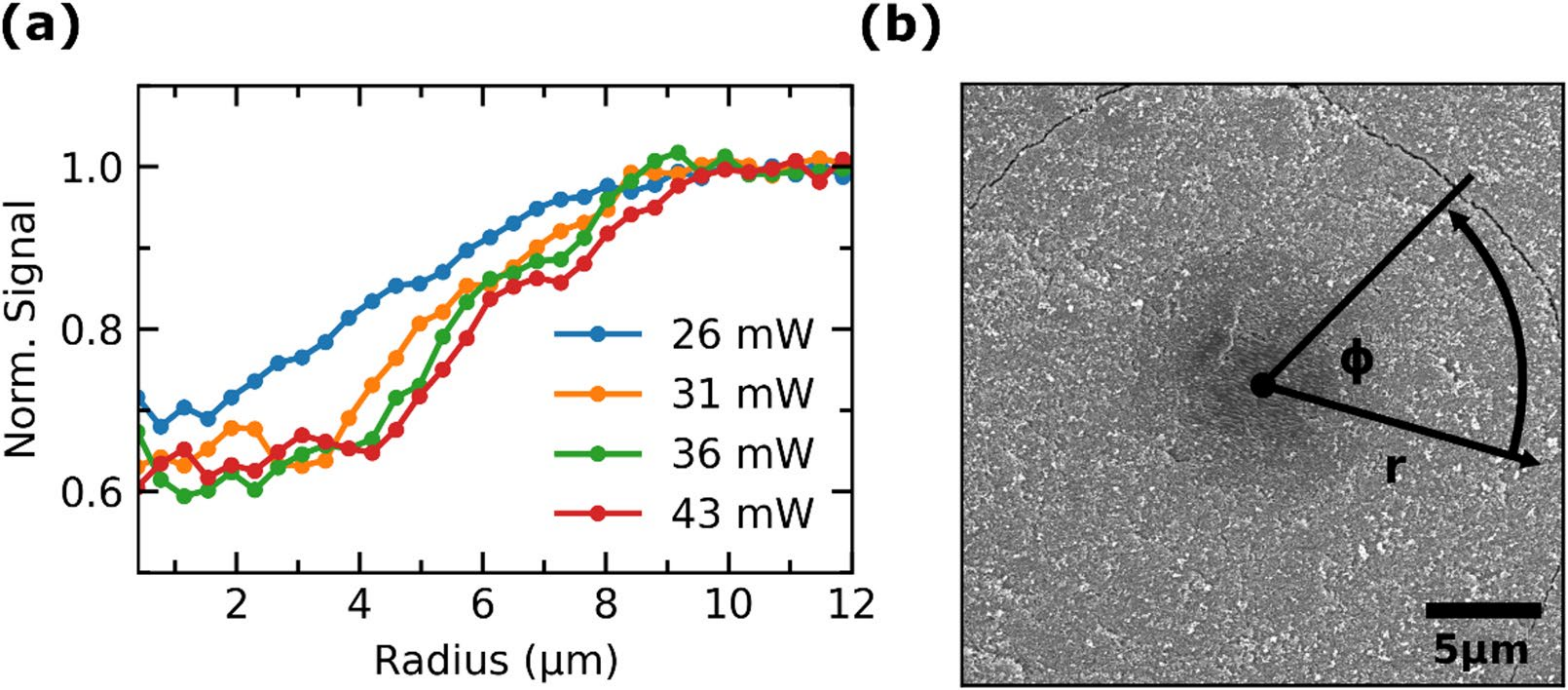
(a) Radial profiles resulting from azimuthal integration of the SEM images (Fig. 6). The signal of the secondary electrons is integrated pixelwise, starting from the middle of the sintering spot. After integration a binning with a bin size of 10 pixels is applied. (b) Visualization of the azimuthal integration. Marked are the radius r and the azimuth angle φ.

The pixel values of the SEM images were azimuthally integrated from the center of the sintering spot (Fig. 7) to quantify the spatial extent of the sintered area and to compare the resulting sintering profile with the measured temperature profile. The integration is performed pixelwise by summing up all the pixel values with a certain distance to the center and dividing it by the number of pixels.

After integration, a binning with a bin size of 10 pixels is applied and the values are normalized to the constant level of the unsintered particles at the edges of the images.

In the SEM images, the average pixel value in the sintered areas is lower due to the reduced specific surface area as compared to the nanoparticulate, porous green body. The larger surface area leads to a greater effective generation volume of the detected secondary electrons^40^, resulting in a higher signal strength. Additionally, the densified area has a higher conductivity compared to the unsintered particular green body. As a consequence, the unsintered area is more likely to be charged by the electron beam, which also leads to a higher signal strength. Hence, in this case, the azimuthally integrated signal strength is used as a qualitative measure of densification. Fully densified material has a lower signal than a porous green body, assuming that no other structural characteristics of the sample or physical effects of secondary electron generation overlap with the signal strength.

The resulting radial profiles exhibit similar spatial characteristics to the measured temperature profiles. The highly sintered area has an extent in the range of the laser beam diameter at power levels from 31 mW to 43 mW (radius of around 4 μm). At a power of 26 mW, the highly sintered area is smaller than the laser beam diameter, which is also evident in the extent of the steep temperature gradient seen by the NIR emission. In the area around the temperature hotspot, a gentle slope of the signal strength is observable. This is likely the area that is not directly heated by absorption of the laser beam, but rather through heat transfer within the material. Due to the low thermal conductivity of the unsintered, nanoporous material and the short process time, densification is limited to an area with a radius of 10 μm. This is about twice the size of the radius of the laser beam, which aligns with a finite element simulation of the laser sintering process with nanoscaled powder^12^. Additional SEM images, showing the laser sintering spots for different laser powers at a higher magnification are in the supplement [Fig. S. 14.]. Furthermore, in the supplement are SEM images of the cracks formed at 43 mW [Fig. S. 15] as well as the microstructural changes at the edge of the spot.

## Conclusion

A commercially available high-speed camera is modified to measure space (< 10 μm) and time (< 1 ms) resolved temperature maps via NIR imaging during resonant laser sintering of a nanoparticulate TiO_2_ sample. The laser sintering temperature is determined from the emitted NIR intensities by a direct camera-sample calibration. The NIR camera system is calibrated for a TiO_2_ sample within the temperature range of 873 K to 1,173 K at a framerate of 1,069 fps. Applying this calibration, the saturation range (70% of full well capacity) of the detector starts at 1,249 K. The exposure time and the color filter may be modified accordingly, if other temperature ranges are measured. At a higher sintering temperature (*T* > 1,249 K), spatially resolved NIR images of resonant laser sintering are recorded at a framerate of 15,969 fps.

The combination of a microscopic lens system and the high-speed camera enables spatially resolved NIR imaging of the heating profile of the focused laser beam (diameter ≈ 9 μm) used for resonant sintering, which is difficult to achieve with mid- or long-wavelength infrared measurements.

The resulting microstructures of the laser sintered TiO_2_ spots are analyzed by SEM. The observed sintering structures agree with the recorded temperature maps and provide suitable laser parameters to achieve full densification.

Due to the small footprint and the stand-alone operating capability of the camera, the developed thermal imaging system is suitable for a wide range of applications, especially in challenging sample environments, where time– and space-resolved high temperature measurements are needed. In such cases, the integration of the imaging system into the measurement set up can be realised with relatively low effort. Additional potential applications that could benefit from this measurement system are in situ X-ray investigations at synchrotron facilities^41–43^, where operando high-speed thermal imaging at high temperatures provides additional insights into the correlation of process parameters and the resulting material characteristics.

## Methods

### Near infrared (NIR) thermal imaging

In the following, the physical basics and consequences for near-infrared thermal imaging are shortly discussed. For a more detailed overview see^27,44^.

Every object at a temperature *T* above absolute zero emits electromagnetic radiation. The radiant exitance *M*_BB_ is strongly dependent on the temperature and for an ideal black body (BB) given by the Stefan-Boltzmann law

1 $${M_{{\mathrm{BB}}}}=\sigma \;{T^4}$$

where *σ* is the Stefan-Boltzmann constant and *T* the absolute temperature^27^. *M*_BB_ describes the radiant power per unit area [W/m^2^] emitted from a surface, independent from direction and wavelength. The spectral radiance *L*_BB_($\lambda ,T$) gives the radiant exitance of a black body per unit wavelength and solid angle [W/m^2^ nm^− 1^ sr^− 1^]. The distribution of the spectral radiance is described by Planck’s law^27^

2 $${L_{{\mathrm{BB}}}}\left( {\lambda ,{\rm T}} \right)=\frac{{2\;h\;{c^2}}}{{{\lambda ^5}\;\left( {\exp \left( {\frac{{h\;c}}{{\lambda \;{k_B}\;T}}} \right) - 1} \right)}}$$

where *h* is Planck’s constant, *c* is the speed of light of the medium in which it is emitted, *λ* is the wavelength and *k*_B_ is the Boltzmann constant. Figure S. 16. shows the spectral radiance of ideal blackbodies at different temperatures between 300 K and 2,000 K. The maximum of the distribution shifts to shorter wavelengths at higher temperatures, as described by Wien’s displacement law. This shift allows for temperature measurements in the NIR region at higher temperatures, i.e. for *T* > 700 K. At lower temperatures, the emitted intensity in the NIR range is too low, especially for measurements at high frame rates and thus short exposure times.

The radiant exitance *M* of a real object is always lower compared to the radiant exitance *M*_BB_ of an ideal black body. The ratio of exitance of a real object compared to the ideal black body is called hemispherical emissivity *ε* and has a value between zero and one.

3 $$M=\varepsilon \;\sigma \;{T^4}$$

The spectral directional emissivity is given by the ratio of its spectral radiance *L* compared to *L*_BB_

4 $$\varepsilon \;\left( {\lambda ,\delta ,\varphi ,{\rm T}} \right)=\frac{{L\;\left( {\lambda ,\delta ,\varphi ,{\rm T}} \right)}}{{{L_{{\mathrm{BB}}}}\;\left( {\lambda ,T} \right)}}$$

Here, *δ* is the zenith angle and *φ* is the azimuthal angle. Aside from the two observation parameters, the wavelength, and the temperature, the emissivity also depends on the material properties of the emitting body. In particular, the surface roughness has a severe influence on the emissivity^27^.

### High speed thermal imaging system

A monochrome Chronos 1.4 high-speed camera (Kron Technologies Inc.)^45^ is used for NIR imaging. The camera features a Luxima Lux 1310 CMOS sensor with 1,280 × 1,024 pixels and a pixel pitch of 6.6 μm. The pre-installed infrared filter is removed to enable NIR measurements. The camera is equipped with a NIR optimized infinity corrected objective (Plan Apo NIR 5x, Mitutoyo) with a magnification of 5 (Fig. 1a).

The lens has a long working distance of 37.5 mm (focal length of 40 mm), which is beneficial for a long depth of focus as well as to provide more space between the lens and allows safe operation in close approximation to a high temperature surface. It is combined with an anti-reflex coated tube lens with a focal length of 200 mm (TTL200-B, Thorlabs) which focusses the infinity corrected NIR radiation to generate a magnified image on the focal plane sensor array. In between the lenses, a high-quality long pass color filter is placed with a cut-on wavelength of 850 nm (FELH0850, Thorlabs). The filter is placed here, in the infinity corrected beam path, to reduce its influence on the beam path. The color filter has an optical density (OD) of more than five for wavelengths shorter than 839 nm, preventing contributions from the visible range and from the UV laser emission. Due to the silicon-based CMOS sensor, the camera is only sensitive to wavelengths shorter than 1.1 μm.

The applied camera has a 32 GB ring buffer, which allows a recording time for 16 s at the highest resolution (1,280 × 1,024 pixel) and frame rate. The data rate in this case is 1.4 Gigapixel per second. The recording length can be increased by using a region of interest (ROI) with smaller pixel number or a decreased frame rate. After recording, the images are exported as raw (DNG) files and processed as NumPy arrays using Python.

**Table 1:** Technical specifications of the Chronos 1.4 high-speed camera which are most relevant for the NIR imaging.

Frame rate	Chronos 1.4 camera (monochrome version)
	1,069 fps at 1,280 × 1,024 px; up to 40,413 at 320 × 96 px
Shutter	global shutter with a minimum exposure time of 1 µs
Dynamic range	56.7 dB; 12 Bit
Ring buffer	32 GB; 16 s recording time at maximum rate of 1.4 gigapixel per second
Sensor	2/3 inch format; 6.6 μm pixel pitch

### UV laser sintering set-up

For sintering, a continuous wave (CW) helium-cadmium gas laser (IK 3102 R-G, Kimmon Koha) with a laser wavelength of 325 nm is used (Fig. 1b). The sample is positioned via a Labview controlled *x*-*y*-*z* microscope stage. The laser beam is guided by dichromatic plane mirrors (041–0325, Eksma Optics) and focused by a plano convex lens with a focal depth of 20 mm (LA4647-UV, Thorlabs) to the sample surface [Fig. S. 4.]. The power of the laser beam is adjusted with a partially transparent, reflective neutral density filter with continuous attenuation (NDC-100 C-2 M, Thorlabs) and is measured by a high sensitivity thermal sensor (Ophir, Model 3 A) behind the focus lens well below the depth of focus. A single blade shutter (SH05, Thorlabs) with the shutter controller (SC10, Thorlabs) is applied to generate defined laser pulses with the CW laser. This combination enables the generation of pulses with a minimum duration of 10 ms.

The camera system is mounted on a micro-adjustment rail combined with a 360° gimbal tripod head, to achieve flexible, yet precise positioning of the camera system (Fig. 1a). The camera is positioned at a zenith angle of 45° to the sample surface normal and the camera focus and the laser focus are adjusted for thermal imaging of the laser sintering.

### Sample preparation and characterization

Nanocrystalline titania (TiO_2_) powder (Aeroxide P25, Degussa) is ground thoroughly in a mortar. The resulting powder is uniaxially, bidirectionally pressed with a pressure of 80 MPa for 5 min and a slow release of the pressure to minimize artifacts in the pellet such as cracks. About 70 mg of nano powder is used, for preparing a cylindrical pellet with a diameter of 1 cm. The pistons are covered by Kapton foil to obtain a smooth and even surface of the resulting green body, which can be easily removed from the pistons. The thickness of the pressed pellets is about 300 μm, which is measured with a laser distance sensor (optoNCDT ILD1220-10, Micro-Epsilon). From this, a green body density of around 40% can be concluded.

The crystallite size of the particles is determined by X-ray diffraction. The diffractogram [Fig. S. 17] is measured with a Rigaku Smartlab with a CBO-µ optics and a 2D detector. A 2*θ* / *θ* measurement is performed of a compacted pellet. The analysis by Rietveld refinement, using Profex^46^ revealed a bimodal crystallite distribution of 26 nm anatase crystallites and 38 nm rutile crystallites. The phase amount of anatase is 88% and of rutile 12%.

After laser sintering at different spots on the TiO_2_ pellet, the resulting microstructures are characterized using a SEM (JSM-7500f, JEOL).

### Temperature measurement principle and calibration

Thermal cameras have focal plane array (FPA) sensors, in contrast to pyrometers which typically measure with only one single active area. The emitted infrared radiation is focused onto the sensor array plane through a lens, and the intensity is measured pixelwise. The result is a spatially resolved array of temperature-dependent intensity levels normalised to the pixel area. The intensity is then converted into temperature by calibration, typically performed using a black body which is precisely temperature-controlled and has an emissivity close to one. The Sakuma-Hattori function is often used as calibration function^47^, fitted to the measurement data using a least-squares algorithm.

The normalization of intensity to the pixel area allows temperature measurements of a hotspot if the hotspot is projected onto the detector such that at least one pixel is fully illuminated. Otherwise, the determined temperature will be underestimated. This smallest spatial resolution which is potentially resolvable is referred to as the instantaneous field of view (IFOV). The IFOV is a result of the imaging size of the lens and the pixel size (pitch) of the FPA, in our instrument it is 6.6 μm.

The emissivity of each surface must be specified to determine the corresponding temperature for subsequent temperature measurements of non-blackbody objects. This is a general issue in measuring temperature via emitted infrared radiation, because the emissivity of the sample is not a strict material constant but specifically depends on the surface characteristics^27^ and is often wavelength dependent. Therefore, it is difficult to find reported emissivity values which correspond to the specific measurement object and the measuring conditions. Especially for powder samples the surface is strongly dependent on the processing and thus the emissivity may vary largely. A promising approach to address the lack of emissivity values is by use of multiwavelength (often two-color) thermometry^3,48^. This method has the advantage that measurements become independent of emissivity. However, it requires that the investigated material approximates the emissivity behavior of a grey body^16,17,49^. Another limitation is the need for simultaneous measurements at two different wavelengths, resulting in a higher instrumental effort. The use of a camera with integrated Bayer color filter (standard sensor technology for digital color cameras^50)^ and thus three different wavelength dependent sensor pixels, results in sensitivity limitation to the visible wavelength range, as well as an overall sensitivity loss, since less sensor area is available for each wavelength range. As a consequence, the measuring temperature must be higher, and the exposure time must be longer compared to a single color NIR measurement with a monochrome FPA.

Many metal oxides, such as TiO_2_ are selective emitters and exhibit strongly wavelength dependent emissivities^51^. Additionally, the emissivity values for TiO_2_ reported in literature vary^52^. Therefore, a special sample calibration has to be performed, even for a multi-wavelength measurement, as the emissivity of the TiO_2_ sample is strongly wavelength dependent.

In this study, the NIR camera intensity is calibrated to the temperature using a TiO_2_ pellet, identically prepared as for laser sintering. In order to do so, the sample is heated to the relevant temperature range, and the emitted NIR radiation is recorded with the high-speed camera under similar optical conditions as used in the laser sintering study. The sample is heated by a silicon nitride (Si_3_N_4_) ceramic heating plate (Bach Resistor Ceramics GmbH), as shown in Fig. 1c. The temperature is measured with a type-K thermocouple which is placed in a blind hole in the center of the heating plate directly beneath the sample position. The sample is put on a thin silicon wafer (375 μm) on the heating plate. The silicon wafer is chosen due to the relatively high thermal conductivity and high flatness of the wafer, which leads to a good thermal contact between the sample and on the other side to the heating plate. The electrical power is controlled by a PID controller with a thyristor power controller, which results in a stable and uniform power supply. Additionally, the apparent surface temperature of the sample is measured by a pyrometer (Optris CT 3MH3, optic CF4, λ = 2.3 μm). Thermal insulation by glass wool reduces cooling due to convection. The housing has an opening above the heating element to enable the optical measurements of the surface and to prevent reflections on the sample surface. The camera is mounted on an optical rail and the zenith angle of incidence is set to 45°.

Intensity images *I*_*x,y,t*_ *(T)* of the heated sample are taken over a temperature range from 773 K up to 1,173 K, in 100 K increments. The images are recorded, when the pyrometer and the thermocouple shows that the temperature of the sample has stabilized and reached a constant value (approximately 10 min after setting the temperature, see [Fig.S. 1.]). For each temperature, 100 images are taken with a constant frame rate of 1,069 fps and an exposure time of 929 µs. The internal darkfield (DF) correction of the Chronos 1.4 camera is used *(I*_*x,y,t*_ *- DF*_*x, y*_*)*. From the resulting images, a region of interest (ROI) of 400 × 500 pixels, located in the center is selected. From these hundred ROIs, collected at a single temperature, first the individual pixels are temporally averaged < *I*_*x,y,t*_*(T)-DF*_*x,y*_ >, and then spatially averaged, over all resulting pixels:

5 $$I\left( T \right)=\overline {{\left\langle {{I_{x,y,t}}\left( T \right) - D{F_{x,y}}} \right\rangle }}$$

Due to the used microscope optics, there is a non-uniform pixel response (NUPR), which is dominated by the vignetting effect^53^. The vignetting leads to a decreasing intensity towards the edges of the detector. This undesired gradient in the intensity can be corrected using a flatfield (FF) correction. For determination of the flatfield, a polished silicon wafer, which has a highly uniform surface, is heated up to 850 K with the same setup to achieve a uniform illuminating area on the camera. Hereof, 100 images are taken with an increased exposure time of 4 milliseconds, to achieve a sufficient digital level (DL) for all pixels, despite a lower emitted intensity of the wafer compared to the TiO_2_ sample. The flat field is used to correct the NIR intensity images, following

6 $${I_{\left( c \right)x,y}}\left( T \right)=\frac{{{I_{x,y}}\left( T \right) - D{F_{x,y}}}}{{\left\langle {F{F_{x,y,t}} - D{F_{x,y}}} \right\rangle }}F{F_{max}}$$

The result is a temperature dependent intensity level which is used for calibration by the Sakuma-Hattori equation. The Planck form of the Sakuma-Hattori equation is given by

7 $${I_{\left( c \right)}}\left( T \right)=\frac{C}{{\exp \left( {\frac{{{c_2}}}{{AT+B}}} \right) - 1}}$$

Herein *A*,* B* and *C* are constants determined by least-squares fitting, which depend on the individual intensity response of the imaging system, whereas *c*_2_ = 14.388 µK is the second radiation constant^47^.

## Supplementary Information

Below is the link to the electronic supplementary material.

Supplementary Material 1

## Data Availability

The datasets used and/or analyzed during the current study are available from the corresponding author on reasonable request.
