# Supplementary material for: Operando high speed near infrared imaging during laser sintering of nanoparticles for time and space resolved temperature measurements
Source: Sci Rep. 2026 Mar 3;16:8158. doi: 10.1038/s41598-026-37445-7 (PMC12960817; doi:10.1038/s41598-026-37445-7)
Supplement: Supplementary file 1 — Supplementary Material 1 [file 41598_2026_37445_MOESM1_ESM.docx]

**Supplementary material**


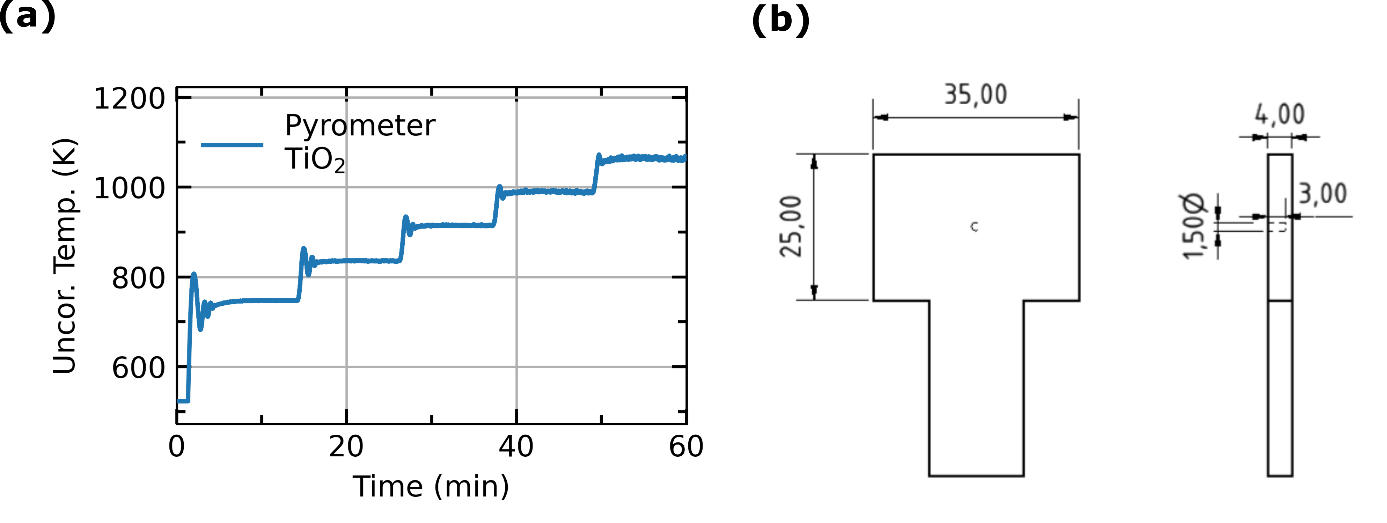
*FIG. S. 1* (a) The plot shows the uncorrected apparent temperature (*ε* = 1) of a TiO₂ sample measured by a pyrometer (Optris CT 3MH3, optic CF4, *λ* = 2.3 µm) during the calibration process. Since the emissivity of the TiO₂ sample is lower than 1, the actual surface temperature is higher than the apparent temperature. The heating system setpoints are 773 K, 873 K, 973 K, 1,073 K, and 1,173 K, measured by a type-K thermocouple inserted into a blind hole of the heating plate. In (b) are the dimensions (given in millimeters) of the Si_3_N_4_ heating plate and the position of the blind hole presented. The NIR images for calibration are recorded with the high-speed camera once the sample temperature has stabilized, which is approximately 10 minutes after setting the setpoint.


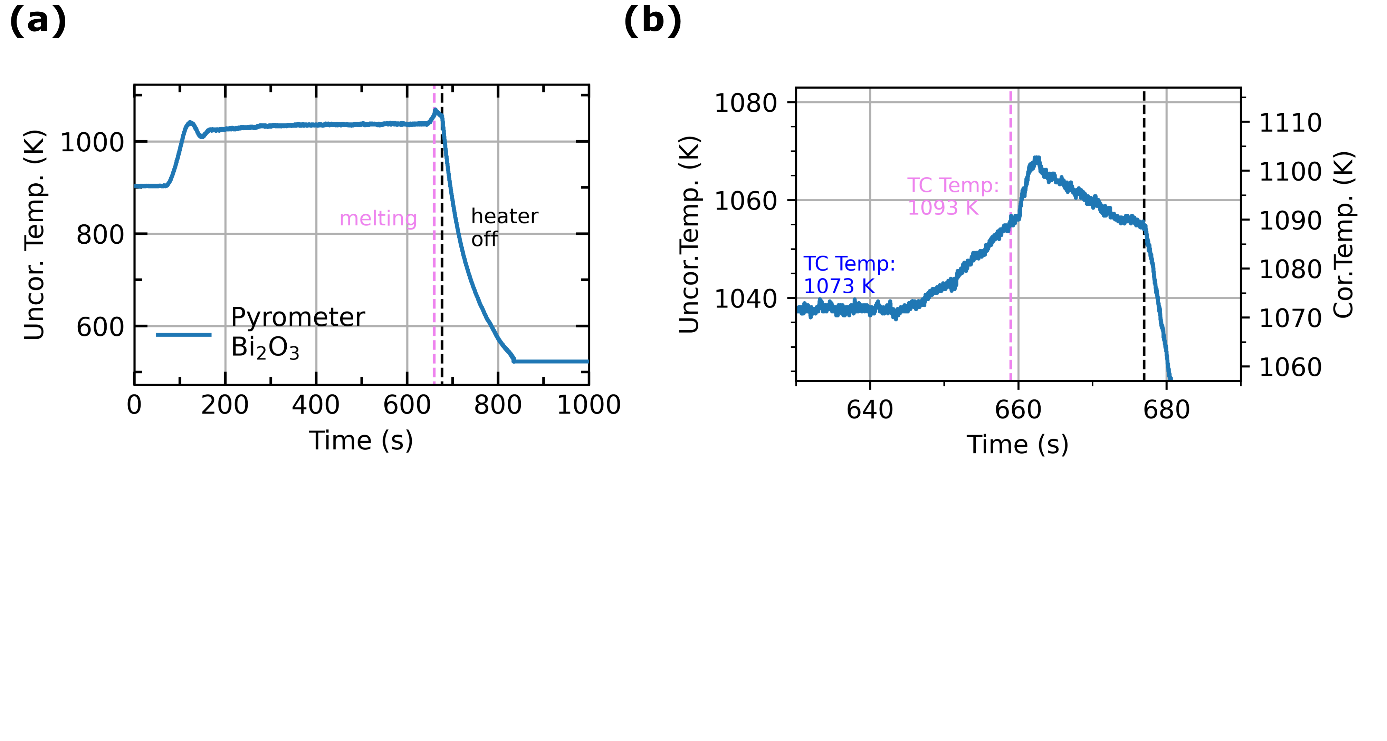
*FIG. S. 2* **(a)** Shows the temperature–time profile of a Bi₂O₃ pellet, which is prepared and heated in the same manner as the TiO₂ pellet. The figure presents the apparent temperature (*ε* = 1) measured by the pyrometer. The heating element is initially set to a temperature of 1,073 K. After the temperature stabilized, the setpoint was increased to 1,098 K. When the thermocouple indicated a temperature of 1,093 K, the pellet melted completely and spread over the surface of the silicon wafer. **(b)** Illustrates the moment of melting with expanded temporal resolution. The apparent temperature exhibits a short plateau (the point in time is marked by a dashed violet line), presumably caused by the melting process. Subsequently, the apparent temperature shows a sudden increase followed by a slight decrease, even though the heating element continued to supply heat before being turned off (indicated by the black dashed line). The corrected temperature (34 K offset) measured by the pyrometer is shown on the right-hand axis, along with the thermocouple readings.

In conclusion, the deviation of the sample temperature and the measured temperature of the thermocouple is in the range of uncertainty of the thermocouple.


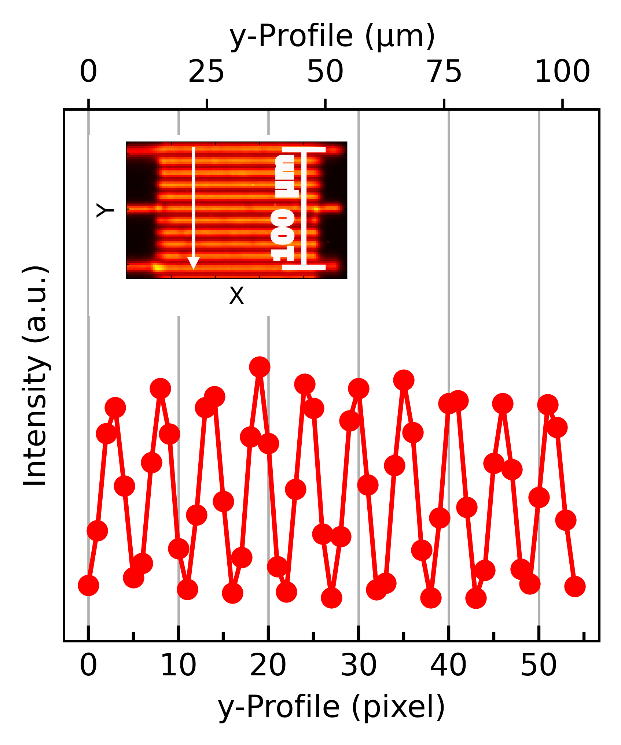


*FIG. S. 3* An Olympus microscope calibration scale is illuminated with a tungsten light bulb, and NIR images are captured with a zenith angle of 45°. The distances of the scale bars provide a resolution of 10 µm. The figure shows a magnified NIR image of the scale bar and the corresponding intensity profile of the reflected NIR radiation. The experiment reveals, that the IFOV of 1.34 µm per pixel of the imaging system is compressed vertically by the factor $\sqrt{2}$ when the zenith angle is kept at 45°. The determined vertical resolution of the NIR microscopic camera (*m* = 5x, pixel pitch = 6.6µm) is 1.90 µm/px under these conditions.


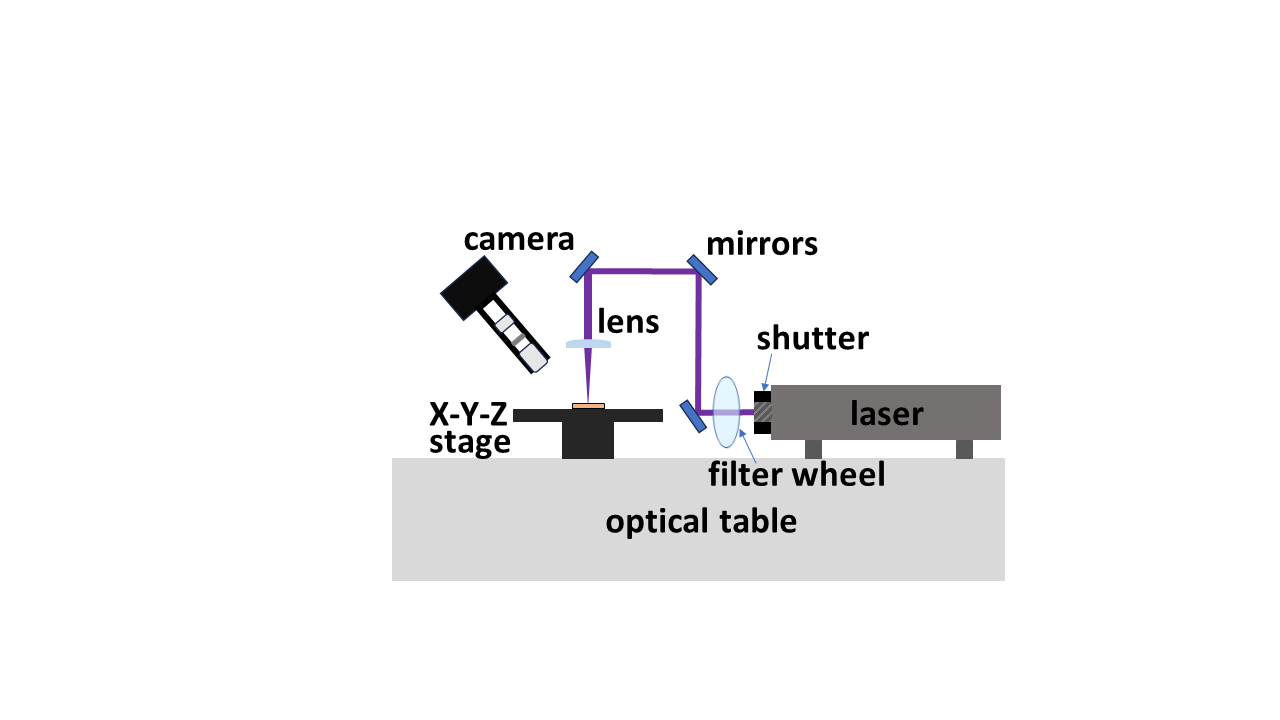


*FIG. S. 4* Schematic of the laser sintering setup. It shows the optical components and indicates the laser beam path. A continuous-wave Helium-Cadmium gas laser (*λ* = 325nm, maximum output power *P* = 100 mW) is used for sintering. The laser beam is guided by dichromatic plane mirrors and focused by a plano convex lens with a focal depth of 20 mm to the sample surface. The *x*-*y*-*z* microscope stage is electronically actuated and controlled via LabVIEW. An electronic single-blade shutter is used to generate laser pulses with a minimum pulse length of 10 ms.


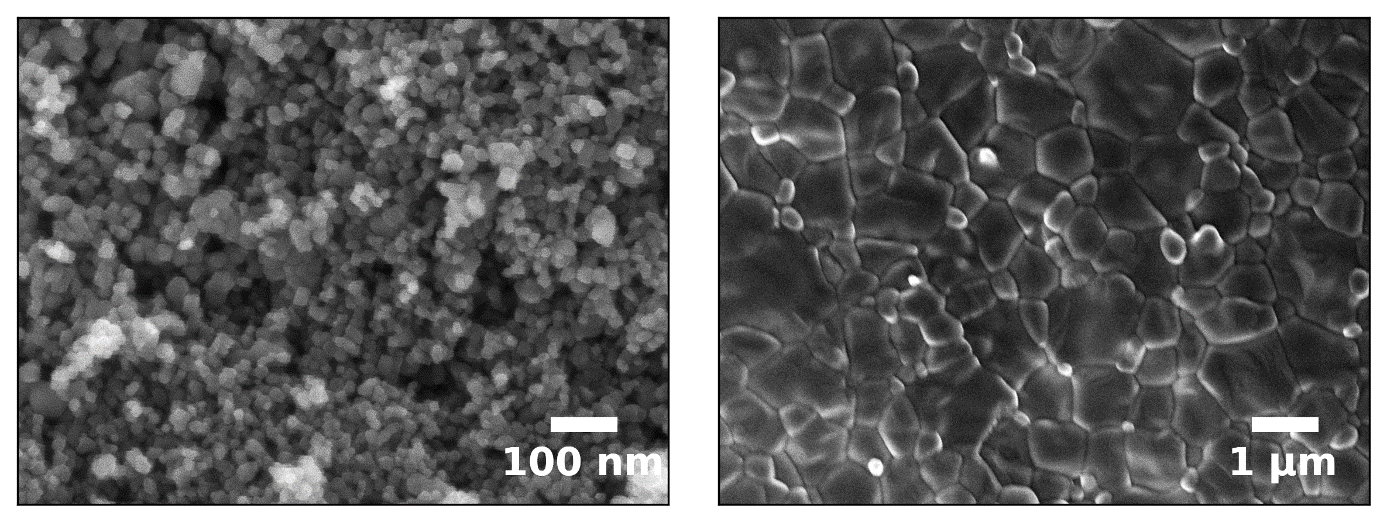


FIG. S. 5 **(Left)** SEM image of the TiO_2_ sample after compaction (magnification 100,000) and **(right)** SEM image of the calibration sample after the calibration process (magnification 10,000). The sample was heated using the temperature-time profile shown in [Fig. S. 1a]. The images depict the microstructural evolution during the sintering process by densification and grain growth.

The following experiments are conducted to determine whether the applied long-pass filter is sufficient to suppress all fluorescence and diffusely reflected laser light. Resonant laser sintering exhibits a threshold in power density at which sintering and a pronounced temperature increase occur, whereas fluorescence and reflections do not. Therefore, the focusing lens was slightly adjusted so that the focal plane was positioned just above the sample surface. This modification alters the power density at the surface without changing the overall laser power. Accordingly, no NIR emission occurs, and no intensity should be measurable with installed long-pass filter in the camera. Additionally, a fluorescence card (VRC1, Thorlabs) is irradiated with a laser pulse, to produce a high fluorescence signal. The intensity curves recorded with the high-speed camera are compared to intensity curves measured by a photodiode. For this a photodiode (Osram SFH 203 P) and a microcontroller (Arduino Uno R4, clock speed 48 MHz) are used in a reverse bias circuit for fast response. The photodiode is read out by the Arduino at a frequency of 25 kHz. The diode is placed close to the irradiation point (~1 cm) at an incidence angle of about 45 degrees, except for the measurement of the direct beam, where the diode is placed in the laser beam before the focus lens. The intensities of the photodiode are filtered by a digital Savitzki‑Golay‑Filter for noise reduction.

**
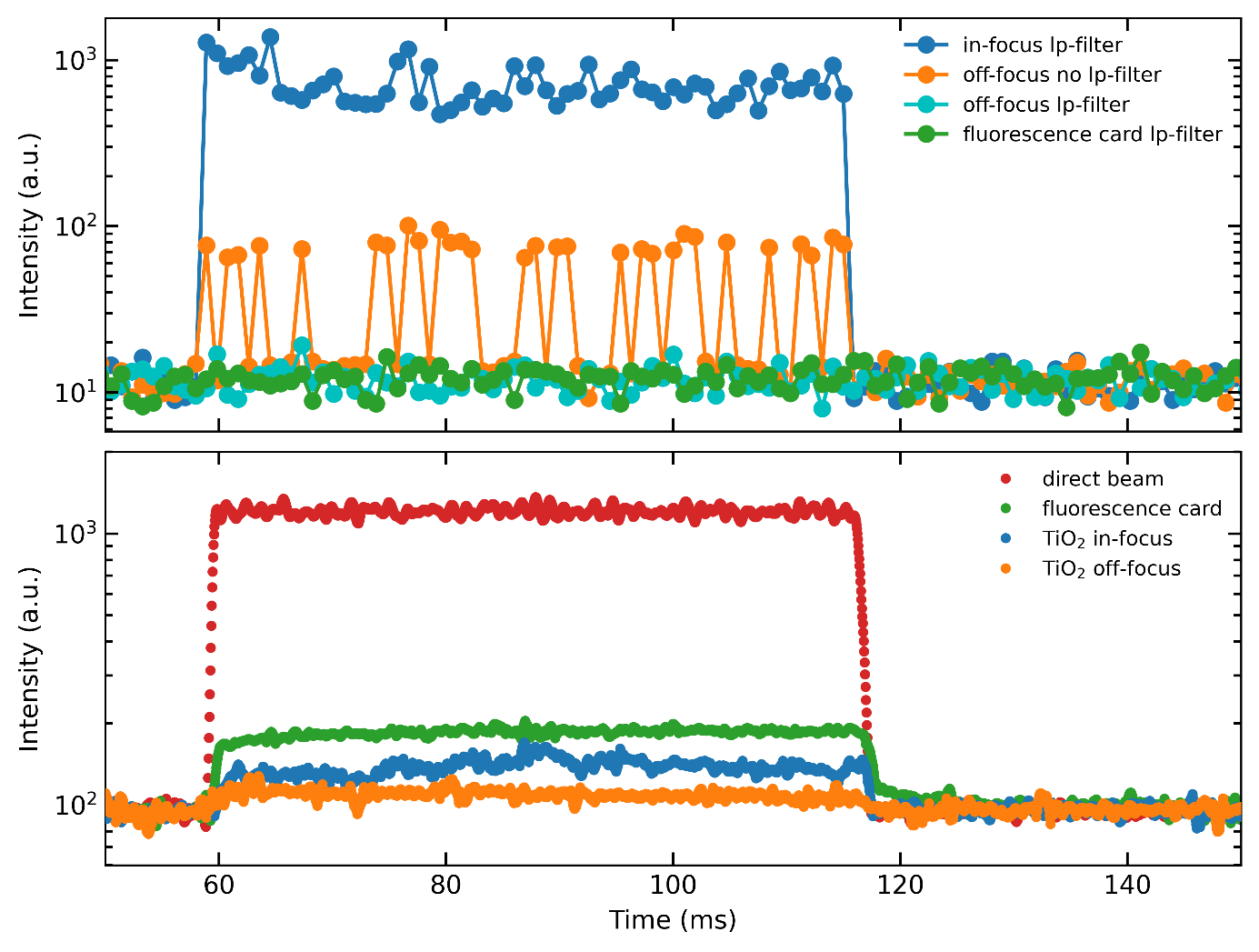
**

FIG. S. 6 The **upper** graph shows the intensity of these UV laser irradiation processes which are recorded with the high-speed camera. The blue curve represents the measured intensity of an irradiation of TiO_2_ (laser power: 45 mW) while the sample surface is in the focal plane of the laser beam, as in the other described laser sintering processes. The high intensity is caused by the NIR emission due to the heating of the sample. The orange curve shows the intensity of an irradiation of the TiO_2_ sample when the lens, focusing the laser beam is moved a few microns upwards using a piezo stage. As a consequence, the focal plane is slightly above the sample surface, and the power density is reduced. Due to the lower power density, the sample is not heated sufficiently (< 873 K). For the orange-colored curve, the long-pass filter is removed from the camera. Therefore, the recorded intensity may originate from reflections of the laser beam or fluorescence of the sample. The cyan-colored curve shows the intensity of an irradiation with the same conditions but with installed long-pass filter (850 nm). Due to the high optical density of the filter (> OD5), the intensity during the irradiation is the same as before and after the irradiation (dark noise). The green curve shows the measured intensity when irradiating a fluorescence card (Thorlabs VRC1) with installed long-pass filter, which effectively suppresses the resulting light. The **lower** graph depicts the photodiode intensities. The red curve shows the intensity when the photodiode is placed directly in the laser beam. The green, blue and orange curve are corresponding to the irradiation processes of the graph above. The signal of the photodiode shows that the irradiation of the fluorescence card produces the highest overall intensity compared to the in- and off-focus irradiation of the TiO_2_ sample. These three irradiation experiments reveal, that the long pass filter is sufficient for suppressing any fluorescence of the sample and diffuse scattering of the laser beam that may occur.


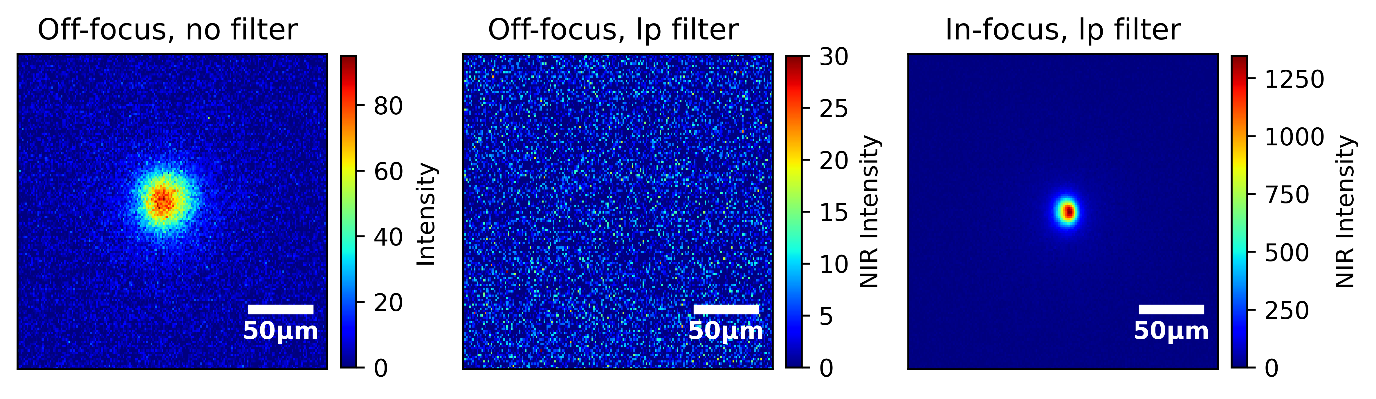


FIG. S. 7 illustrates false color images of irradiations of a nanoparticulate TiO_2_ sample. The images are recorded with a framerate of 1,069 fps at the beginning of the laser pulse. The y-axis of the images is stretched by a factor of $\sqrt{2}$ to compensate for geometric distortion due to the zenith angle of 45°. To obtain a rectangular image, fewer pixels are displayed in the y-direction than in x-direction. **(Left)** The focus lens of the laser beam is moved a few microns upwards, so that the focal plane is slightly above the surface of the sample. This leads to insufficient heating (< 873 K) due to the lower power density. The recorded intensity might come from fluorescence or diffuse scattered laser radiation, as no color filter is inserted to the camera. **(Middle)** The image depicts the start of a laser pulse with identical conditions, but a 850 nm long pass filter is inserted in front of the camera. Only dark noise is visible. **(Right)** The image shows the NIR Intensity when the laser focal plane is at the surface of the sample and a 850 nm long pass filter is inserted in the camera system. Due to the higher power density the sample is heated more intensely and emits NIR radiation.


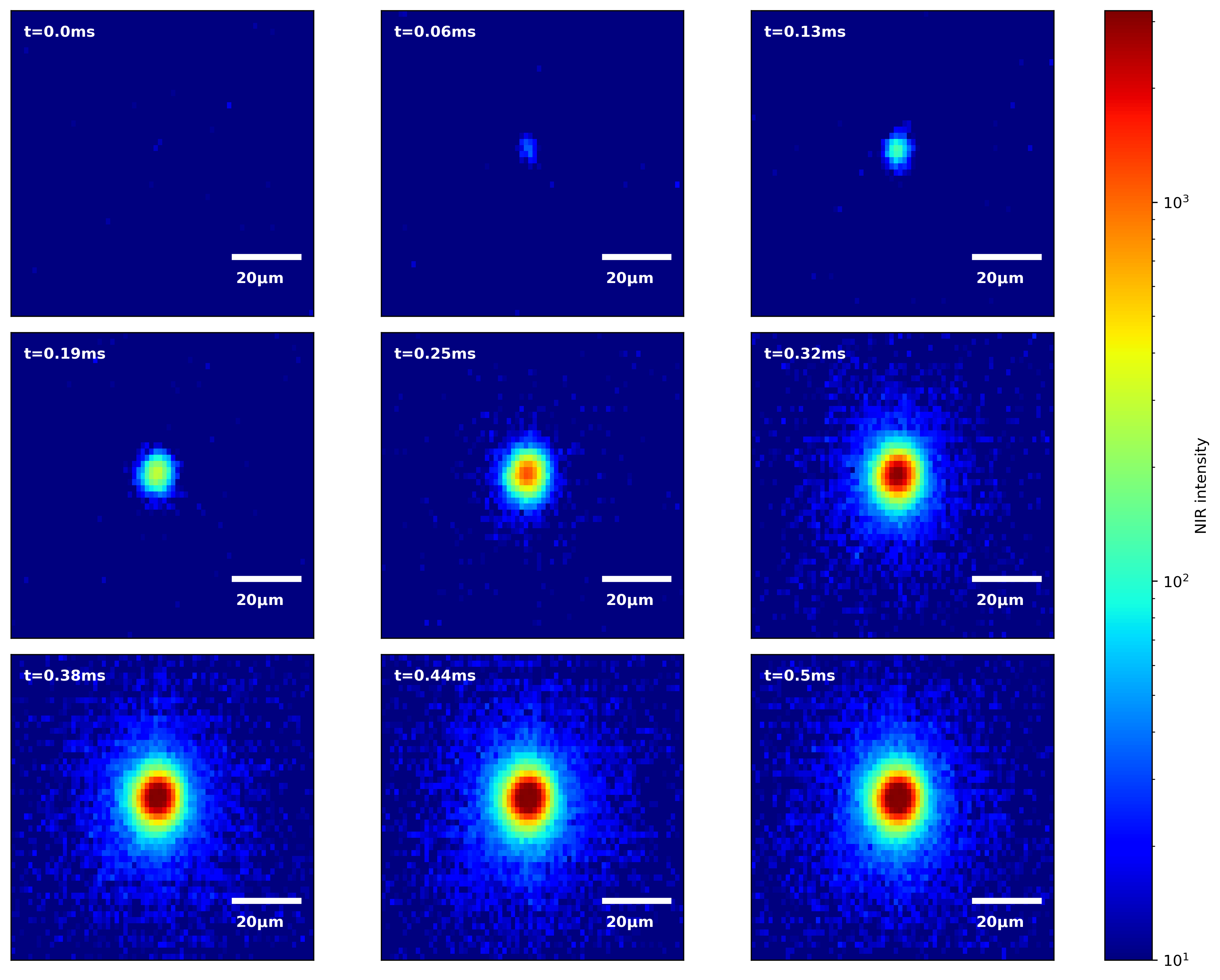
FIG. S. 8. NIR images of laser sintered TiO_2_ nanoparticles with a laser power of 65 mW and a pulse duration of 50 ms, recorded operando at a frame rate of 15,969 fps. The development of the emitted NIR radiation shows the rapid temperature increase generated by the local and resonant absorption of the laser beam. The y-axis of the images is stretched by a factor of $\sqrt{2}$ and fewer pixels are displayed in y-direction than in x-direction to compensate for geometric distortion due to the zenith angle of 45°.


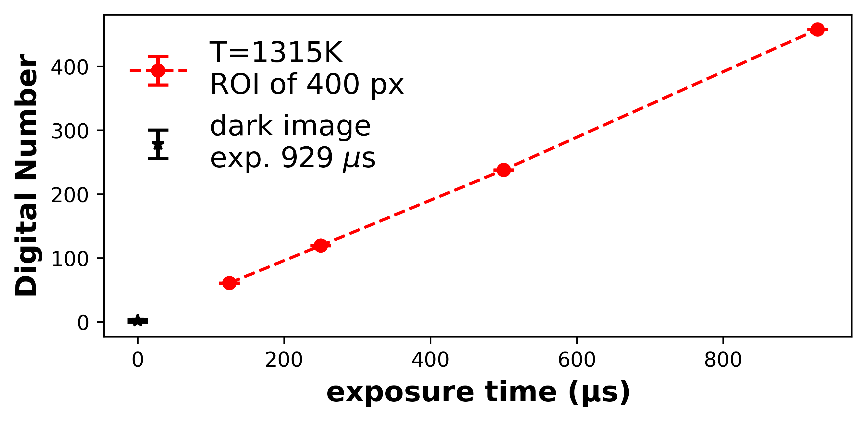


FIG. S. 9 exhibits the dependency of the signal (Digital Number DN) from the exposure time of the Chronos 1.4 high-speed camera. In this experiment a tungsten filament in a light bulb is used as stable heat source. The temperature of 1,315K is determined by the temperature dependent resistance of the filament. The images are recorded with a bandpassfilter (Thorlabs, FBH930-10) with a center wavelenght of 930 nm. For the analysis a ROI of 400 px, where the tungsten filament has a plane and uniformly emitting surface, is chosen. The averaged digital numbers of 30 frames each reveal the strong linear dependency from the exposure time. The errorbars represent the standard deviation of the ROIs. The black star shows the averaged digital number of 30 dark frames, which are recorded with an exposure time of 929 µs. For all images, the internal dark correction of the camera is used, leading to the low digital number of dark noise.


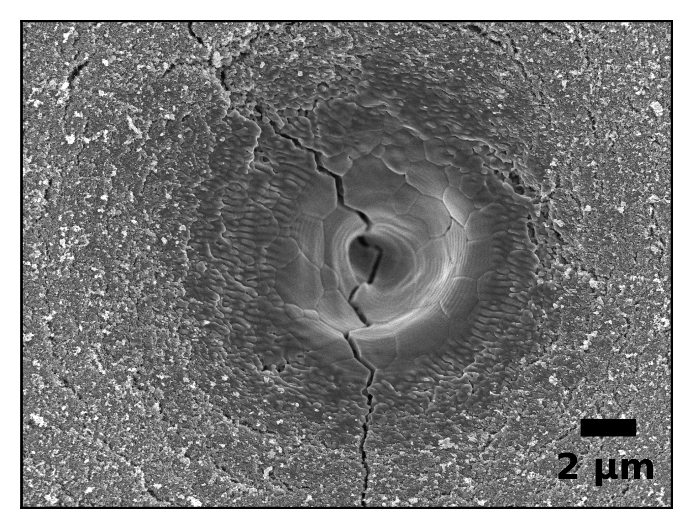


FIG. S. 10. SEM image of the nanoparticulate TiO_2_ sample after laser sintering with a laser power of 65 mW and a pulse duration of 50 ms.

**
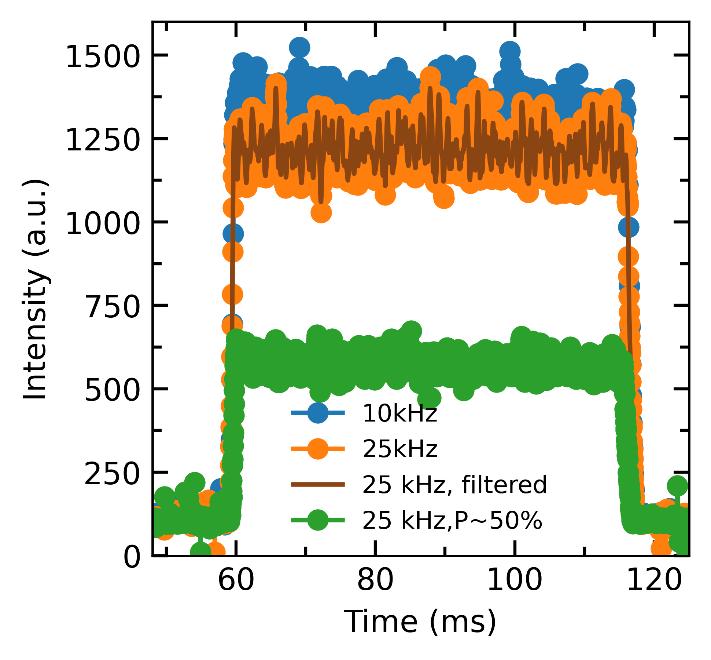
**

*FIG. S. 11* The graph shows the intensity profile of a 50 ms laser pulse. The intensity is recorded using a photodiode (Osram SFH 203 P) and a microcontroller (Arduino Uno R4, clock speed 48 MHz) in a reverse-bias configuration to ensure a fast response. The photodiode is selected for its relatively high sensitivity in the UV region and its fast rise and fall time of 5 ns. The orange curve shows the signal when the photodiode was placed directly in the laser beam. The sampling rate is 25 kHz (period 40 µs), limited by the analog readout speed of the Arduino. The brown curve is from the same laser pulse, but the intensity is filtered by a digital Savitzki‑Golay‑filter. The blue curve represents an identical 50 ms pulse recorded at a sampling rate of 10 kHz. A comparison of both profiles reveals the high repeatability of the pulse durations provided by the beam shutter (see Methods). The green curve shows the intensity profile of a 50 ms laser pulse at approximately 50 % reduced laser power.


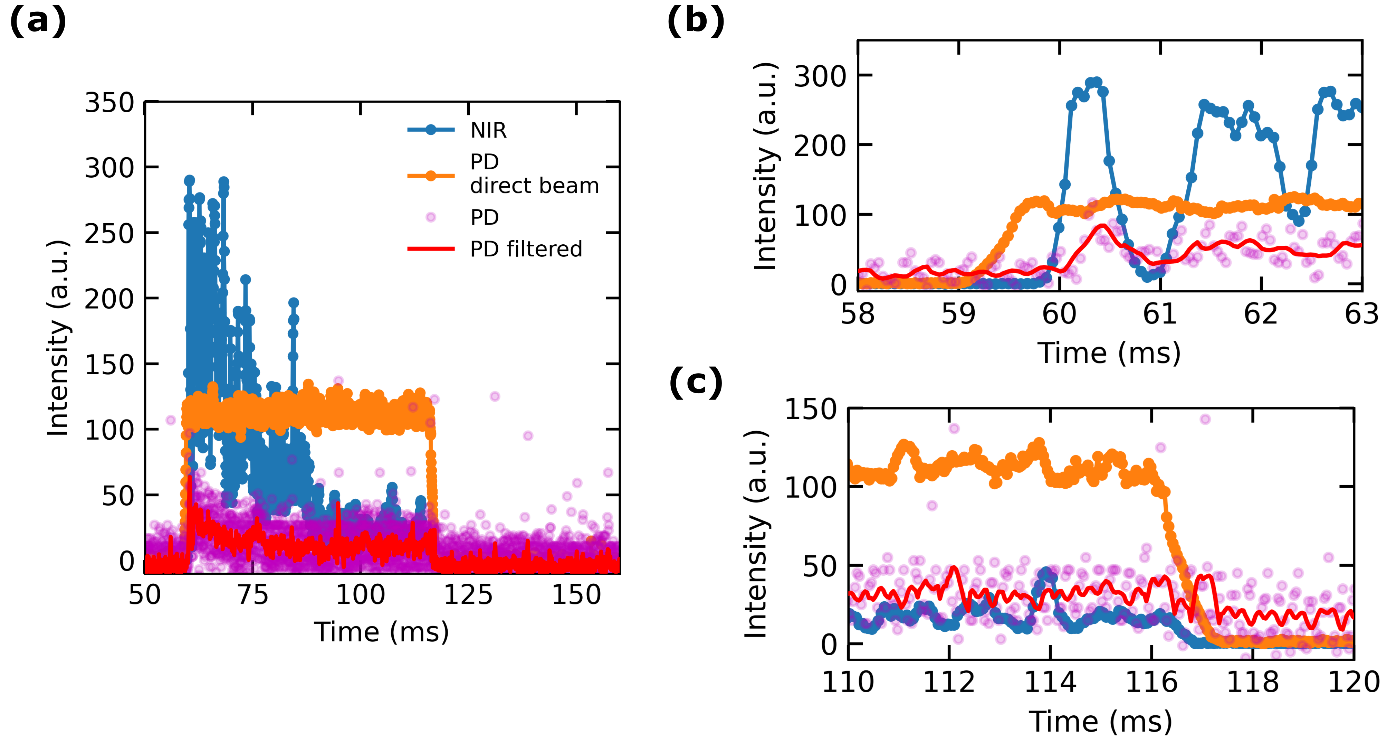
*FIG. S. 12* (**a)** presents the NIR intensities recorded by the high-speed camera at a frame rate of 15,696 fps during a laser sintering process of TiO₂ with a laser power of 61 mW. In addition, the corresponding photodiode (PD) intensity profile (recorded at frequency of 25 kHz) of the same process is shown, together with the photodiode signal of the direct beam from a 50 ms laser pulse. For better clarity, the NIR intensity and the direct beam intensity are scaled down by a constant factor, the photodiode dark noise is subtracted, and the Savitzky–Golay filtered photodiode signal is displayed in front of the unfiltered intensity. Figure (b) shows a time-expanded view of the beginning of the laser pulse, and (c) a time-expanded view of the end of the laser pulse. The signal of the photodiode (red) which is directed to the sintering spot, has a delay compared to the signal of the direct laser beam. The delay is probably caused by the low signal level of the photodiode. The signal only exceeds the background noise when NIR radiation is emitted by the sample due to the temperature increase.


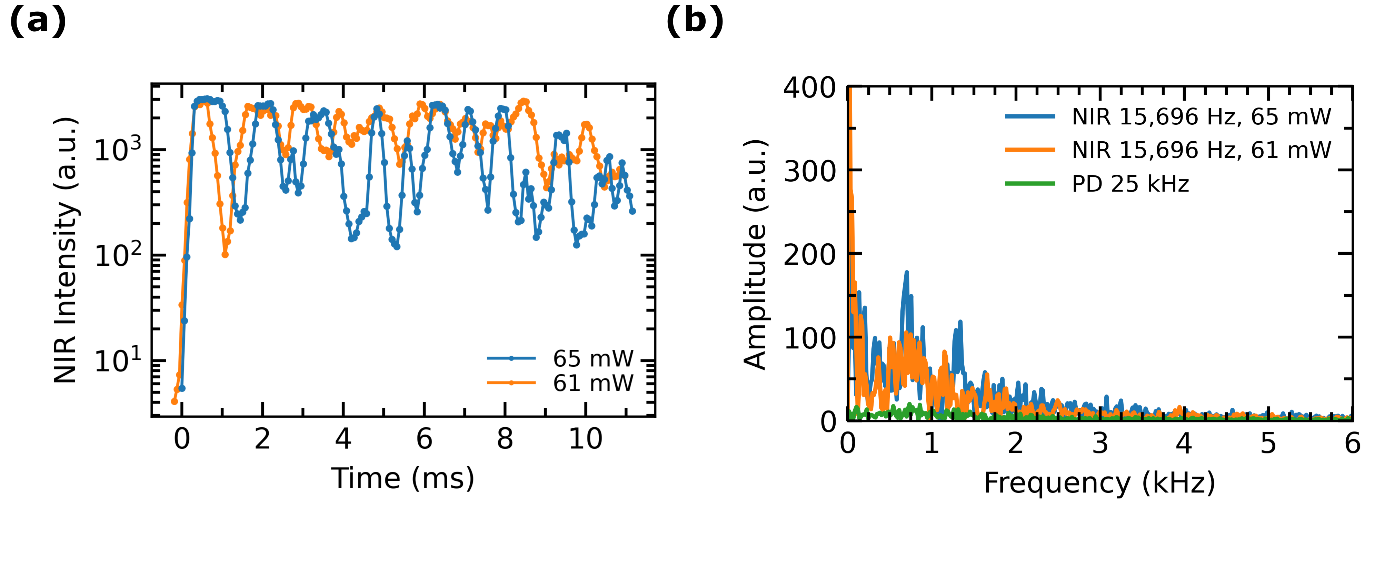


*FIG. S. 13* In (a) is shown the NIR intensity of two laser pulses on TiO_2_. The figure presents a time magnified view of the beginning of the laser pulse, where the recorded intensity is the highest. The NIR images are recorded at a frequency of 15,696 fps. Figure (b) depicts the Fast Fourier Transformation (FFT) of the intensities shown in (a). Additionally, the FFT of the signal of the photodiode (PD) of the direct laser beam is shown. The figures reveal that there is no direct correlation between the oscillations in the measured NIR signal and in the much smaller intensity fluctuations of the laser beam.


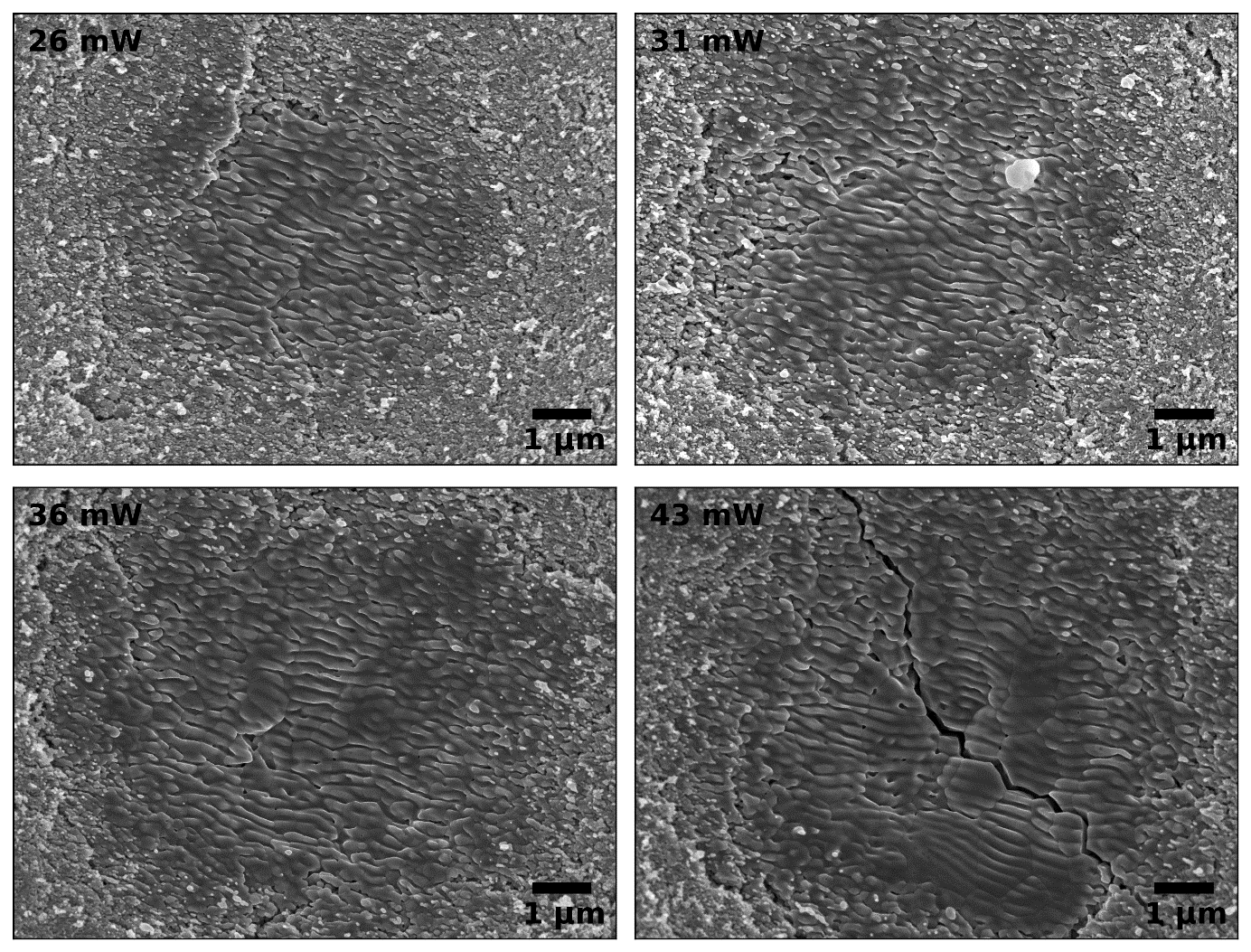


FIG. S. 14 SEM images (magnification of 10,000) of the sintering spots at the surface of the TiO_2_ powder bed after laser irradiation with different laser powers.


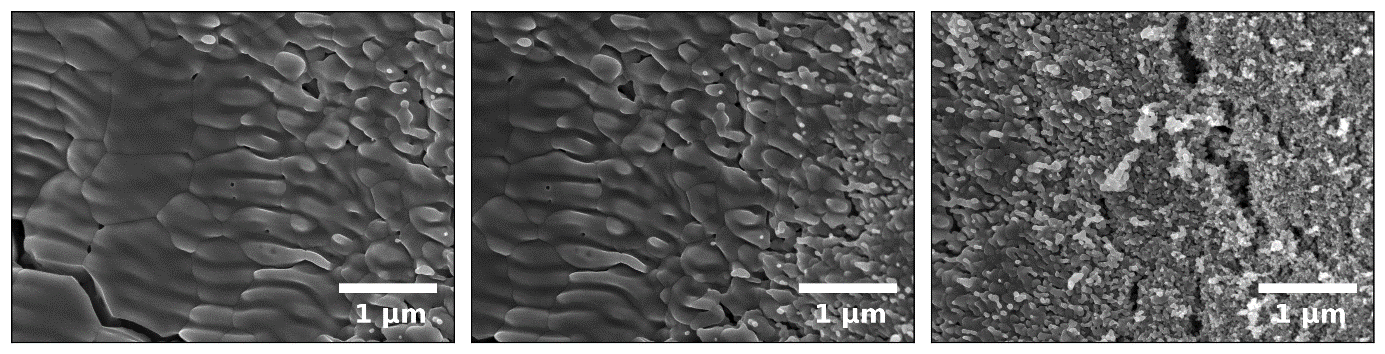


FIG. S. 15 SEM images (magnification of 25,000) of the sintering spots at the surface of the TiO_2_ powder bed after laser irradiation with 43 mW. The images depict the peripheral region of the sintering spot. Due to the steep temperature gradient, a pronounced change in the microstructure occurs within a range of a few microns.


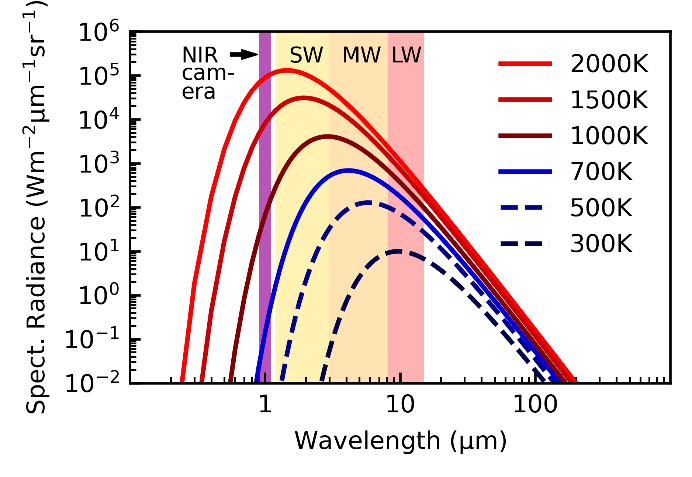


FIG. S. 16 Spectral radiance of a blackbody, L_BB_(λ,T), for increasing temperatures. The wavelength range of the used NIR imaging system (NIR camera) is highlighted in magenta. The system is sensitive for T ≥ 700 K (solid lines). The SW, MW, and LW ranges of the infrared region are highlighted in addition.


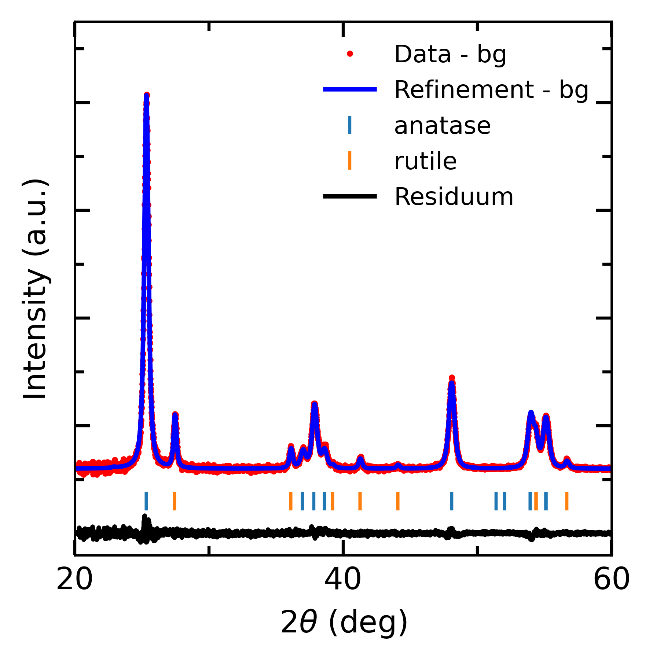


*FIG. S. 17* The figure depicts the X-ray diffraction (XRD) data of the used TiO_2_ nanoparticles. The particles are compacted into a pellet (see Methods). The diffractogram is measured by a Rigaku Smartlab with a CBO-µ beam optics in a 2 θ / θ measurement with a 2D detector. The Rietfeld Refinement is performed with Profex [46] and the peak shape of the instrument is fitted using a LaB_6_ reference measurement. The refinement of the XRD data reveals that the TiO_2_ particles are a mixture of anatase (88%) and rutile (12%). The crystallite size of the anatase phase is around 26 nm and the rutile crystallite size is around 38 nm.

**Evaluation of uncertainty**

The uncertainty of the measured intensity is evaluated by:

$\sigma_{I}=\sqrt{\left( \frac{s}{\sqrt{N_{Pixel}}} \right)^{2}+\left( \left| \frac{dI}{dT} \right|\sigma_{T_{TC}} \right)^{2}}$ , where *s* is the experimental standard deviation, *N*_Pixel_ is the number of Pixels in the recorded frames, and *σ_T_* is the measurement uncertainty of the thermocouple (class 2 type-K thermocouple, 0.0075∙T).

As in Figure 3b the values of the pixels of one image row are shown, the *N*_Pixel_ differs. Accordingly, the *N*_Pixel_ is one and in Figure 3c and 3d is a ROI of *N*_Pixel_ = 9 used. Finally, the uncertainties of the temperatures are evaluated by:

$\sigma_{T}= \sqrt{\left( \frac{dT}{dI} \sigma_{I_{t}} \right)^{2}}= \left| \frac{dT}{dI} \right|\sigma_{I_{t}}$, where. $\sigma_{I_{t}}=\sqrt{\left( \frac{s}{\sqrt{N_{Pixel}}} \right)^{2}+\left( \left| \frac{dI}{dT} \right|\sigma_{T_{TC}} \right)^{2}}$

The calculated standard deviation *s* for the calibration points shows an exponential increase with temperature in the calibration, therefore the standard deviation values for the laser sintering temperatures are obtained with an exponential fit from these.
